# Histidine Ethylation by Histidine Methyltransferases SETD3 and METTL9

**DOI:** 10.1002/cbic.70403

**Published:** 2026-06-17

**Authors:** Jordi C. J. Hintzen, Zhimei Yu, Sadaf Ahmad, Xin Zhang, Yuan–Yuan Zhao, Hao Deng, Leonie Schütz, Marija Ram, Julia Z. Kaminska, Jakub Drozak, Elmar Weinhold, Hong Guo, Andrea Rentmeister, Ping Qian, Jasmin Mecinović

**Affiliations:** ^1^ Department of Physics Chemistry and Pharmacy University of Southern Denmark Odense Denmark; ^2^ School of Chemistry and Chemical Engineering Liaoning Normal University Dalian P. R. China; ^3^ Chemistry and Materials Science Faculty Shandong Agricultural University Tai’an P. R. China; ^4^ Institute of Organic Chemistry RWTH Aachen University Aachen Germany; ^5^ Department of Chemistry Ludwig‐Maximilians‐Universität München Munich Germany; ^6^ Department of Metabolic Regulation Faculty of Biology University of Warsaw Warsaw Poland; ^7^ Department of Biochemistry and Cellular and Molecular Biology University of Tennessee Knoxville Tennessee USA

**Keywords:** biochemistry, chemistry, histidine, imidazole, methylation, regioselectivity

## Abstract

AdoMet‐dependent histidine methyltransferases catalyze regioselective methylation of histidine residues in proteins. N^τ^‐Methylation of His73 in β‐actin is catalyzed by histidine methyltransferase SETD3, and represents a unique post‐translational modification involved in the regulation of actin polymerization. Likewise, N^π^‐methylation of His375 in zinc transporter SLC39A5 is catalyzed by histidine methyltransferase METTL9, thereby modulating zinc‐binding properties of SLC39A5. Here, we report biomolecular studies on the ability of human SETD3 and METTL9 to catalyze the histidine ethylation reaction beyond methylation. Combined synthetic, biocatalytic and computational analyses employing synthetic or in situ formed AdoMet analogs AdoEth and AdoSeEth reveal that AdoMet is the most efficient cosubstrate; however, SETD3 and METTL9 also have the capacity to catalyze ethylation of histidine in β‐actin and SLC39A5 peptides, respectively. Computational analyses support the experimental observations and provide the structural origin for more efficient histidine methylation than ethylation reaction. This work provides an insight into the molecular requirements for histidine methyltransferase‐catalyzed histidine methylation and most related ethylation reactions on the N^τ^‐ and N^π^‐positions in the imidazole ring, the knowledge important for functional assignment and design of chemical probes targeting histidine methyltransferases.

## Graphical abstract

Enzyme assays and computational analyses reveal that human histidine methyltransferases SETD3 and METTL9 have a capacity to catalyze histidine ethylation in the presence of synthetic or in situ produced *S*‐adenosylethionine (AdoEth) and *Se*‐adenosylselenoethionine (AdoSeEth) cosubstrates.

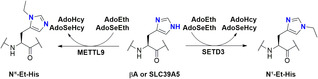

## 1 Introduction

**FIGURE 1:**
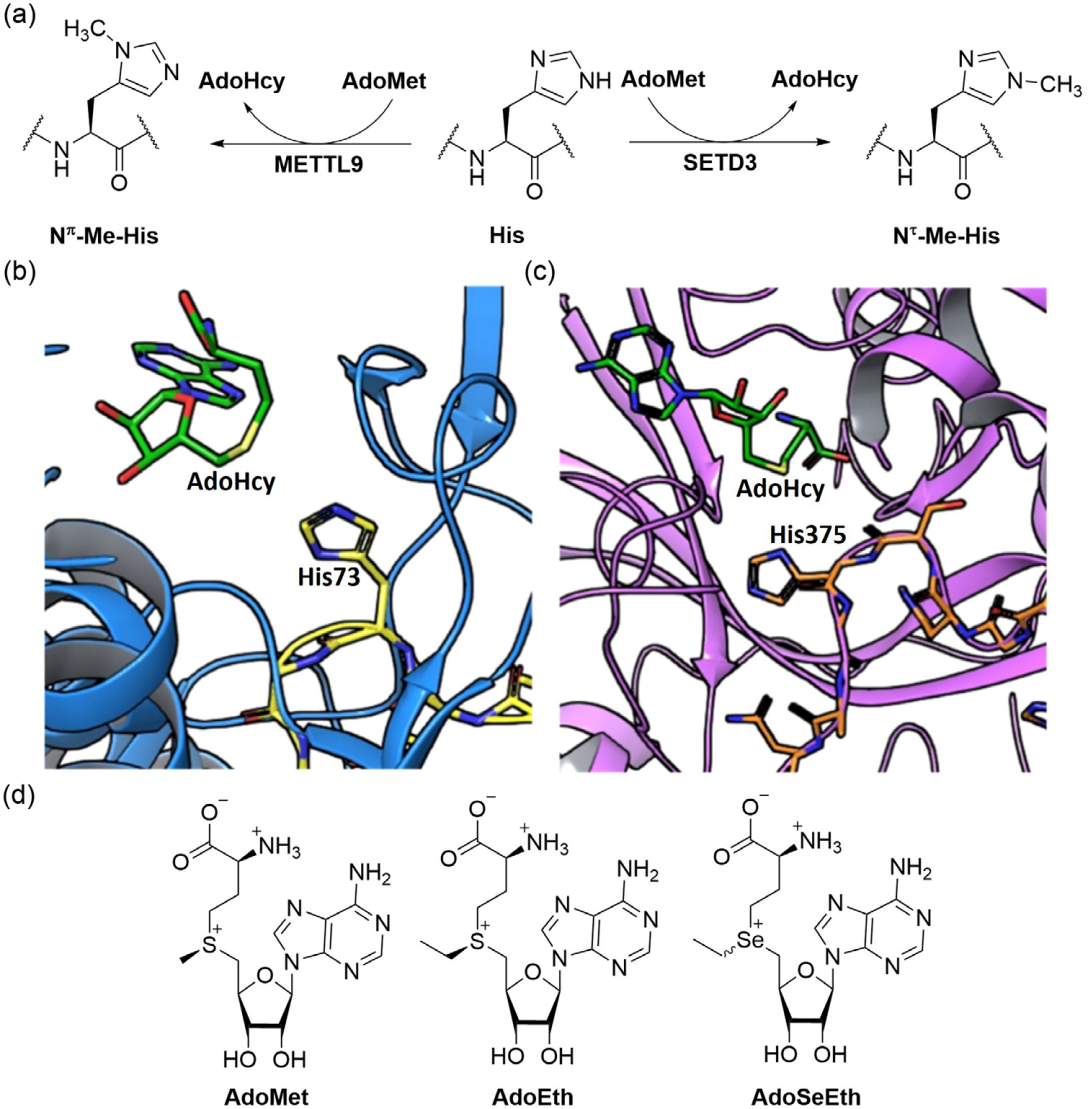
AdoMet‐dependent histidine methylation by histidine methyltransferases. (a) SETD3‐catalyzed N^τ^‐methylation of His73 in βA in the presence of AdoMet and METTL9‐catalyzed N^π^‐methylation of His375 in SLC39A5 in the presence of AdoMet. (b) View from a crystal structure of SETD3 (blue) complexed with the βA‐His73 peptide (yellow) and *S*‐adenosylhomocysteine (AdoHcy, green) (PDB ID: 6ICV). (c) View from a crystal structure of METTL9 (purple) complexed with the SLC39A5‐His375 peptide (orange) and AdoHcy (green) (PDB ID: 7YF2). (d) Structures of AdoMet, AdoEth, and AdoSeEth used in our study.

Histidine methyltransferases have recently gained recognition as enzymes of emerging biomedical relevance, owing to their roles in regulating protein function and associations with human disease [1, 2, 3]. Despite growing biomolecular interest, the substrate and cosubstrate specificity of histidine methyltransferases for methylation of the N^τ^ and N^π^ positions of the histidine's imidazole ring remain underexplored (Figure 1a). Among them, SETD3 and METTL9 represent the best‐characterized examples, each catalyzing site‐specific methylation of histidine residues in distinct protein substrates [4, 5, 6, 7, 8]. SETD3 modifies His73 in β‐actin via N^τ^‐methylation, influencing actin filament dynamics and cytoskeletal organization, whereas METTL9 catalyzes N^π^‐methylation of His375 in zinc transporter SLC39A5 (ZIP5), affecting its zinc‐binding properties [1, 3, 6, 9]. Dysregulation of these enzymes has been implicated in various cancers and other diseases [10, 11], underscoring the biological significance of histidine methylation as a widespread post‐translational modification (PTM) [12]. However, the full (co)substrate scope, catalytic plasticity, and physiological relevance of these enzymes remain incompletely understood.

Actin is an important protein in the regulation of the cytoskeleton in eukaryotic cells, as it is a constituent of the microfilaments, playing an important role in the cell's structural stability [13]. It exists in six different isoforms in humans, among which β‐actin (βA) is ubiquitously expressed [14, 15]. β‐Actin undergoes extensive PTMs, which can influence the stability of the actin filaments and therefore, are involved in many cellular processes [9]. Among these modifications is the N^τ^‐methylation of His73 that has been linked to an increased stability of the actin filaments through a decreased rate of the ATP hydrolysis needed for disassociation of the monomers [16]. SETD3 is the enzyme that has recently been identified to be responsible for βA‐His73 methylation [4, 5, 11], although it was initially found to catalyze methylation of lysine residues at positions K4 and K36 of histone H3 [17]. SETD3 is a member of the SET domain containing methyltransferases [18], a superfamily of enzymes that typically catalyze methylation of arginine and lysine residues in numerous proteins, with SETD3 being the exception due to a unique histidine‐specific methyltransferase activity (Figure 1) [19]. SETD3 has the capacity to methylate histidine mimics incorporated in the βA peptide [20]. Interestingly, human SETD3 was found to be inhibited by the βA peptide possessing methionine and its analogs at the His73 position, which led to the identification of several nanomolar inhibitors of SETD3 [21]. Moreover, the G74S mutation of βA is believed to be responsible in part for causing Baraitser–Winter cerebrofrontofacial syndrome, and the effect of the G74S mutation on the SETD3 methylation activity was recently studied [22]. Furthermore, the importance of the Ile71 and Trp79 secondary binding pockets was explored by incorporation of isoleucine and tryptophan analogs into synthetic βA peptides, respectively, [23, 24]. The results of these studies were then helpful in the identification of α‐centractin (ACTR1A), an actin‐related protein, as a new SETD3 substrate that is specifically methylated at its His77 residue [25]. Finally, prestructured *i*, *i + 3* stapled cyclic peptides were used to evaluate the influence of structural flexibility around the His73 of β‐actin residue, demonstrating that SETD3 prefers linear βA substrates [26].

On the other hand, the methyltransferase METTL9 was recently found to catalyze N^π^‐methylation using a conserved His‐x‐His motif [6, 8, 27]. While SETD3‐dependent methylation of β‐actin is specifically localized at His73, methylation by METTL9 was found to be much more promiscuous and found to be pervasive among the human proteome [8]. However, histidine methylation of certain zinc transporters, including SLC39A5 (ZIP5), was found to promote tumor growth, indicating important biological implications for the N^π^‐methylation by METTL9 [6, 8, 27, 28]. A recent study investigated the substrate specificity of METTL9 orthologs and found that while the His‐x‐His target site is required, METTL9 orthologs from different species display different substrate specificities [29]. Moreover, it was recently shown that human METTL9 has an exceptionally narrow substrate selectivity for histidine and does not have the ability to catalyze methylation of histidine analogs [30].

*S*‐Adenosylmethionine (AdoMet, SAM) is the universal cosubstrate used for protein methylation by functionally diverse methyltransferases, producing the *S*‐adenosylhomocysteine (AdoHcy, SAH) by‐product (Figure 1b–d). Analogs of AdoMet have been used to introduce reactive handles onto DNA, RNA, and proteins by AdoMet‐dependent methyltransferases [31, 32, 33, 34, 35, 36, 37], and replacing AdoMet's sulfur atom with selenium has been shown to increase the alkylation reactivity [38, 39]. Bulkier AdoMet analogs have been used to introduce a variety of functional groups into proteins, including click‐reactive handles for protein labeling purposes [40]. Here, we report on the ability of human histidine methyltransferases SETD3 and METTL9 to transfer the ethyl group of AdoEth and AdoSeEth to the histidine substrate of βA and SLC39A5 peptides, respectively (Figure 1d).

## 2 Results and Discussion

### 2.1 SETD3‐Catalyzed Histidine Ethylation

**FIGURE 2:**
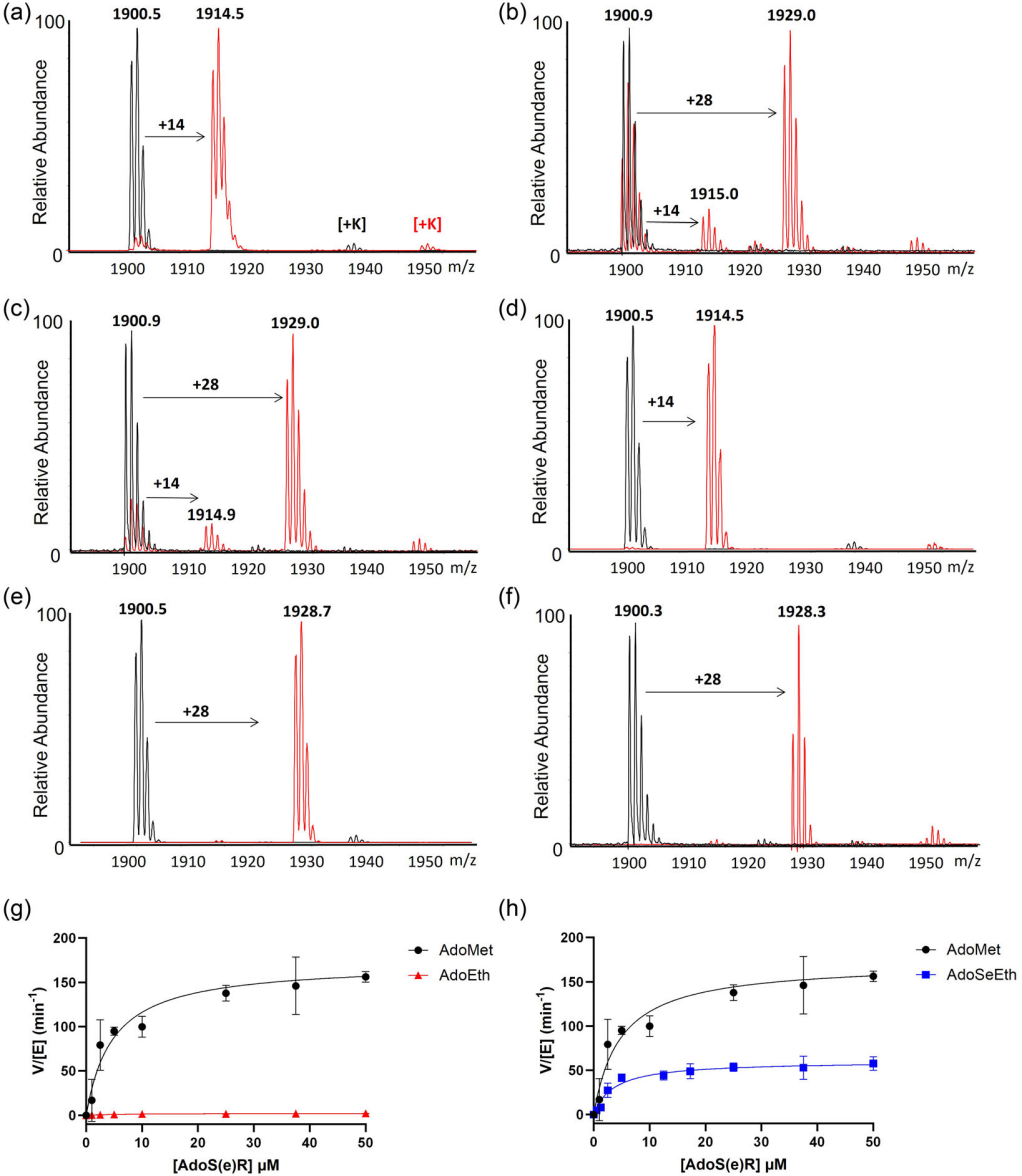
MALDI‐TOF MS data showing alkylation of the βA peptide (10 μM) in the presence of SETD3 (1 μM) and AdoMet and its analogs (100 μM) after a 3 h incubation at 37°C at pH 9.0; (a) AdoMet, (b) AdoEth, (c) AdoSeEth. MALDI‐TOF MS data showing alkylation of the βA peptide (10 μM) in the presence of SETD3 (5 μM) and AdoMet and its analogs (100 μM) after 5 min preincubation and a 3 h reaction at 37°C at pH 9.0; (d) AdoMet, (e) AdoEth, (f) AdoSeEth. Control reactions in the absence of SETD3 are shown in black, SETD3‐catalyzed reactions are shown in red. (g,h) Enzyme kinetic plots of SETD3‐catalyzed methylation and ethylation of βA by AdoMet (black), AdoEth (red), and AdoSeEth (blue). All reactions were run in triplicate, and errors are reported as standard errors (SE).

AdoEth was synthesized using the previously reported protocol [41], while AdoSeEth was synthesized by triflation of ethanol and subsequent alkylation of selenium in *Se*‐adenosylselenohomocysteine (AdoSeHcy) in the presence of a mixture of formic and acetic acid to ensure protonation of nucleophilic positions other than selenium (Figure 1d) [41, 42]. The βA peptide (residues 66–81, TLKYPIEHGIVTNWDD) was synthesized using standard microwave‐assisted solid‐phase peptide synthesis and subsequently purified by reverse phase high‐performance liquid chromatography (HPLC) (Figures S1, S2). Using the synthetic βA peptide, we performed MALDI‐TOF MS‐based enzymatic assays to establish the ability of SETD3 in accepting AdoEth and AdoSeEth as cosubstrates, together with the AdoMet positive control. As a reference, βA peptide (10 μM) was incubated with AdoMet (100 μM) and SETD3 (1 μM) at 37°C, and the histidine methylation was nearly completed (>90%) within 3 h (Figure 2a). Furthermore, the reaction was proven to be dependent on both AdoMet and SETD3 (Figure S3). Using the established standard conditions, we continued with the evaluation of AdoEth and AdoSeEth as cosubstrates for the SETD3‐catalyzed ethylation of βA‐His73. SETD3 efficiently catalyzed ethylation of histidine, with >90% conversion reached for AdoSeEth and 54% conversion by AdoEth after 3 h, with a minor amount of methylation emerging concurrently (Figure 2b,c). To reach an increased level of conversion for AdoEth, we increased the concentration of the enzyme to 5 μM and ran the experiment under otherwise the same conditions. While the expected ethylation could be observed, it was also seen that in all samples a significant methylation peak appeared, suggesting the presence of AdoMet in the purified enzyme, resulting in unwanted methylation (Figures S4, S5). To avoid this issue, AdoEth was first preincubated with SETD3 for 5 min whereafter the βA peptide was added to facilitate the ethyl transfer reaction. Indeed, methylation peaks were significantly reduced in this case, indicating that AdoEth binds to SETD3 when present in excess, outcompeting the residual AdoMet that is bound to the recombinant enzyme (Figure 2d–f). For AdoSeEth and AdoEth, full ethylation was observed under optimized conditions after 3 h (Figure 2e,f), with minor to nonexistent methylation peaks visible.

Enzyme kinetics evaluations of SETD3‐catalyzed methylation and ethylation were then carried out to assess AdoMet and its two analogs that were efficiently accepted as cosubstrates. MALDI‐TOF MS kinetics studies were used under steady‐state conditions, varying the concentration of AdoMet, AdoEth, and AdoSeEth in the kinetic buffer at pH 9.0. The concentration of SETD3 was adjusted to accommodate linear conversion for the cosubstrates in a time window of 20 min at 37°C, with the highest enzyme to substrate ratio used at 0.5, using 0.5 μM of substrate and 0.25 μM of SETD3. Unsurprisingly, the naturally occurring AdoMet was accepted as the best cosubstrate by SETD3, with AdoEth having a 100‐fold loss in cosubstrate efficiency as a result of decreased *k*_cat_ (Figure 2g, Table 1). However, AdoSeEth only showed a decrease in its *k*_cat_/*K*_m_ value by about twofold, due to a lower *k*_cat_ (Figure 2h, Table 1). *K*_m_ values were only found to be moderately different among the cosubstrates tested, remaining within a twofold difference. Here, the difference between AdoEth and AdoSeEth exemplifies the increased reactivity by substitution of sulfur for selenium, in line with results for lysine ethylation [42]. It has to be noted that the *K*_m_ value for AdoSeEth is an upper estimate because an epimeric mixture at the selenium center was used for kinetic analysis.

**TABLE 1:** Kinetic parameters for SETD3‐catalyzed methylation and ethylation of the βA peptide.

Cosubstrate	***k***_**cat**_^***app***^ **,** **min** ^**−1**^	***K***_**m**_^***app***^, **μM**	***k***_**cat**_^***app***^ **/*K***_**m**_^***app***^ ***,*** **μM** ^**−1**^ **min** ^**−1**^
AdoMet	172 ± 7.7	4.7 ± 1.0	36.3
AdoEth	2.2 ± 0.1	6.2 ± 1.1	0.4
AdoSeEth	60.2 ± 3.3	3.6 ± 0.9	16.5

The arginine residue at position 253 is placed within the SETD3 catalytic pocket and is pointing toward the AdoHcy byproduct in the crystal structures, and could therefore potentially interfere with larger ethyl groups to be efficiently transferred to the target histidine in the βA peptide substrate (Figure 1b). However, R253 forms the salt bridge with the carboxylate group of AdoHcy (see Figure 5a below) and may also play an important role in the AdoMet cosubstrate binding. Moreover, R253 may help to generate the near attack conformation for the reaction complex and lower the activation barrier for the methyl transfer. To determine the outcome of the interplay from different factors for the histidine methyltransferase catalysis, we generated two variants of human SETD3, R253A and R253G, using site‐directed mutagenesis. AdoMet and its two analogs were incubated with both SETD3 variants using standard (10 μM βA, 1 μM SETD3, 100 μM AdoMet) and optimized conditions (10 μM βA, 5 μM SETD3, 100 μM AdoMet, 5 min preincubation). Under standard and optimized conditions, the SETD3 variants did not display any activity toward AdoMet, AdoEth, and AdoSeEth (Figures S6–S9), except for the R253G variant, which poorly catalyzed methylation under optimized conditions (Figure S9), indicating that Arg253 is essential for efficient SETD3 catalysis.

### 2.2 METTL9‐Catalyzed Histidine Ethylation

**FIGURE 3:**
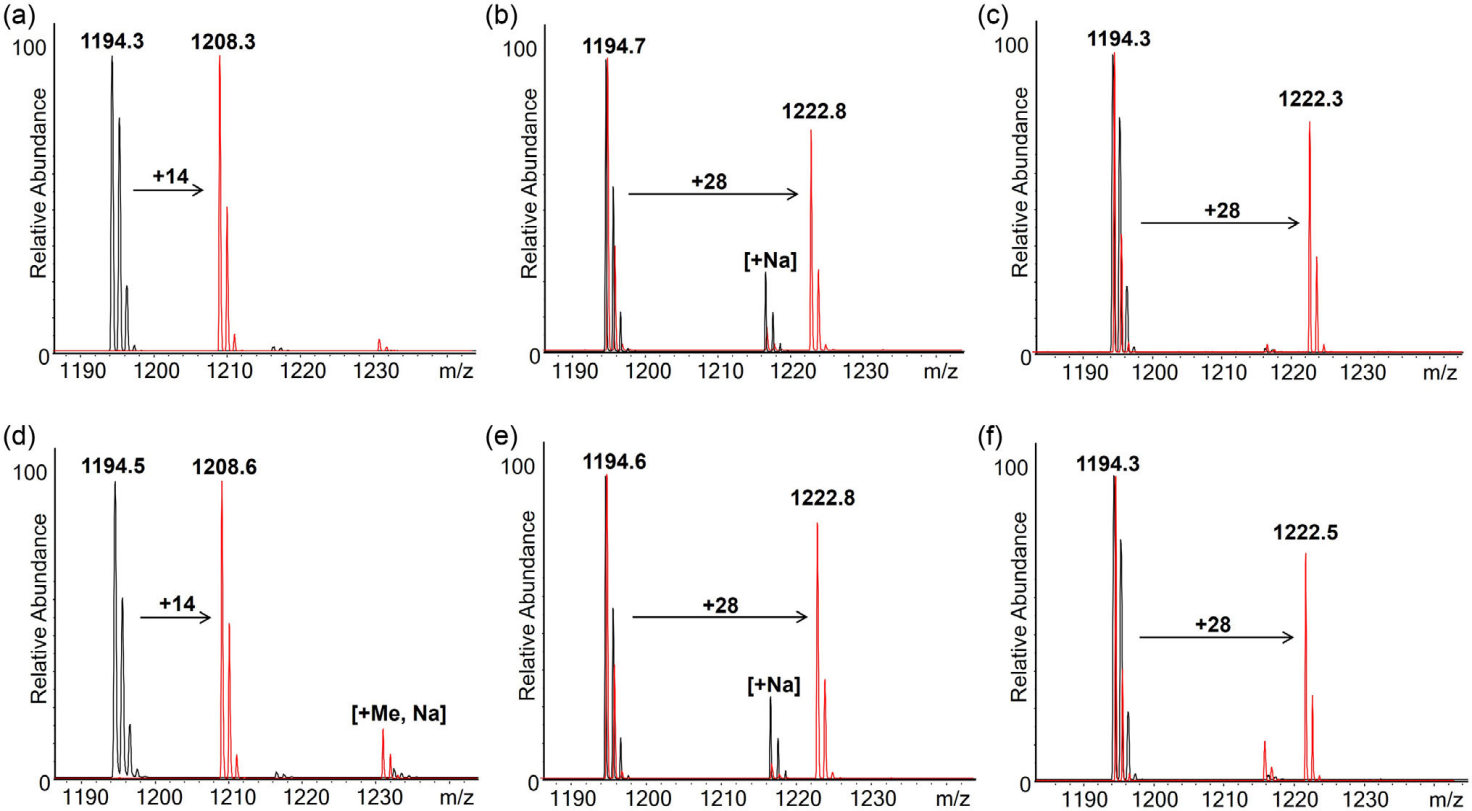
MALDI‐TOF MS data showing alkylation of the SLC39A5 peptide (10 μM) in the presence of METTL9 (5 μM) and AdoMet and its analogs (100 μM) after 5 min preincubation and 3 h reaction. Control reactions in the absence of METTL9 are shown in black, whereas METTL9‐catalyzed reactions are shown in red. (a) AdoMet at pH 7.5 (b) AdoEth at pH 7.5, (c) AdoSeEth at pH 7.5, (d) AdoMet at pH 9.0, (e) AdoEth at pH 9.0, (f) AdoSeEth at pH 9.0.

The SLC39A5 peptide (residues 369–380, GHQGHSHGHQGG) was synthesized using an automated solid‐phase peptide synthesis protocol and purified by reverse‐phase HPLC (Figures S10, S11). Using the synthetic SLC39A5 peptide, we performed MALDI‐TOF MS‐based enzymatic assays to determine the ability of METTL9 in accepting AdoEth and AdoSeEth as cosubstrates. As a positive control, the SLC39A5 peptide (10 μM) was incubated with AdoMet (100 μM) and METTL9 (1 μM) at 37°C at pH 7.5 and 9.0, showing that histidine methylation was completed within 3 h (Figure S12). Likewise, the methylation reaction was proven to be dependent on both AdoMet and METTL9 (Figure S13). Using the established standard conditions, we continued with AdoEth and AdoSeEth. Unlike SETD3, METTL9 did not catalyze ethylation of histidine in the presence of AdoEth and AdoSeEth after 3 h at both pH 7.5 and 9.0 under standard conditions (Figure S12). To ascertain the catalytic ability of METTL9 for the ethylation of histidine, we increased the concentration of METTL9 to 5 µM. Control assays with SLC39A5 (10 µM), AdoMet (100 μM), and METTL9 (5 μM) at 37°C at pH 7.5 and 9.0 showed complete methylation of histidine after 3 h (Figure 3a,d). With AdoEth and AdoSeEth, ethylation was observed with both analogs in the presence of higher concentrations of METTL9. With AdoEth, ~43% and ~46% ethylation was observed at pH 7.5 and pH 9.0, respectively, after 3 h (Figure 3b,e). Similar levels of ethylation were observed with AdoSeEth after 3 h; ~44% ethylation at pH 7.5 and ~41% ethylation at pH 9.0 (Figure 3c,f). These results limit the possibility of obtaining accurate kinetics data for the METTL9‐catalyzed ethylation of SLC39A5.

### 2.3 Histidine Ethylation via Cascade Reactions

**FIGURE 4:**
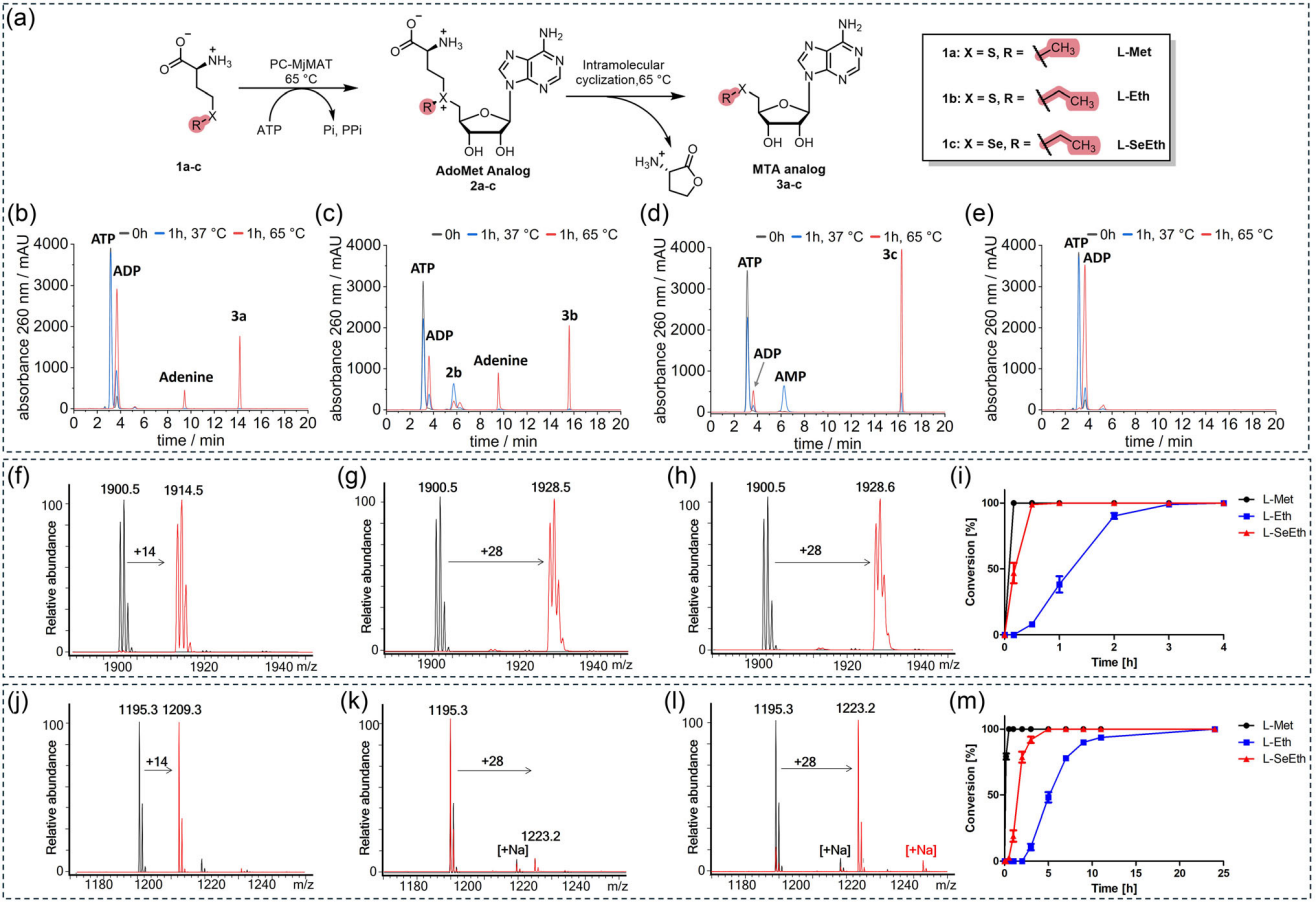
HPLC and MALDI‐TOF MS analyses of cascade methylation and ethylation of histidine substrate residue in βA and SLC39A5 peptides. (a) The MAT reaction scheme showing that L‐methionine, L‐ethionine, and L‐selenoethionine are converted to AdoMet, AdoEth, and AdoSeEth, respectively, followed by an expected decomposition pathway to form **3a–c** at elevated temperature. (b) HPLC analysis of L‐methionine MAT reaction. (c) HPLC analysis of L‐ethionine MAT reaction. (d) HPLC analysis of L‐selenoethionine MAT reaction. (e) HPLC analysis of a negative control reaction. (f) SETD3‐catalyzed methylation of βA with L‐methionine after 3 h reaction. (g) SETD3‐catalyzed ethylation of βA with L‐ethionine after 3 h reaction. (h) SETD3‐catalyzed ethylation of βA with L‐selenoethionine after 3 h reaction. (i) Time‐course for SETD3‐catalyzed methylation and ethylation. (j) METTL9‐catalyzed methylation of SLC39A5 with L‐methionine. (k) METTL9‐catalyzed ethylation of SLC39A5 with L‐ethionine. (l) METTL9‐catalyzed ethylation of SLC39A5 with L‐selenoethionine. (m) Time‐course for METTL9‐catalyzed methylation and ethylation. Error bars are reported as standard deviation for triplicates (*n* = 3; mean ± SEM). Control reactions in the absence of PC‐MjMAT are shown in black, whereas PC‐MjMAT‐mediated reactions are shown in red.

Having shown that SETD3 and METTL9 have an ability to accept AdoEth and AdoSeEth as cosubstrates for histidine ethylation reactions, we next explored a possibility to generate these two cosubstrates in situ using methionine adenosyltransferase (MAT) and carry out a cascade reaction in the presence of histidine methyltransferases [43, 44, 45]. AdoMet analogs with ethyl group replacement were synthesized enzymatically from L‐ethionine or L‐selenoethionine and adenosine triphosphate (ATP) using a methionine adenosyltransferase (MAT) [46]. Here, a recently reported MAT variant from *Methanocaldococcus jannaschii* PC‐MjMAT was used to test the substrate scope using simple amino acids L‐methionine (L‐Met), L‐ethionine (L‐Eth), and L‐selenoethionine (L‐SeEth) (Figure 4). L‐Selenoethionine was synthesized as previously reported [43, 47]. Initially, the PC‐MjMAT activity was evaluated alone with L‐methionine, L‐ethionine, and L‐selenoethionine with ATP as previously described [47]. The formation of AdoMet analogs was monitored by HPLC, based on their ability to undergo intramolecular cyclization to generate methylthioadenosine (MTA) analogs, which produces a detectable signal at 65°C (Figure 4a). A control reaction in the absence of L‐methionine was also included to confirm that product formation was substrate dependent (Figure 4b–e). The results indicated that all the substrates gave good conversions, with L‐selenoethionine leading to nearly quantitative formation of AdoSeEth. Then, one‐pot assays were performed using the recently reported protocol [44]; 25 µM PC‐MjMAT, 1 mM ATP, 2 mM L‐methionine, L‐ethionine, or L‐selenoethionine, 5 µM SETD3, and 100 µM βA peptide in a pH 8.0 buffer system. AdoEth and AdoSeEth were generated in situ and immediately utilized by SETD3 for histidine ethylation via a one‐pot cascade reaction. Reaction progress was monitored by MALDI‐TOF MS at different time points. The full conversion of the βA peptide was observed after 30 min using L‐methionine and L‐selenoethionine, whereas reaction with L‐ethionine required a longer time (3 h) (Figure 4f–i). Additionally, to gain a better understanding of the reaction progression and primary time‐course of the histidine ethylation in the presence of L‐ethionine, timepoints were taken for the SETD3‐catalyzed ethylation reactions, showing almost full conversion after 2 h (Figure S14). The βA‐His73et product was confirmed by MS/MS (Figure S15). The MAT‐METTL9 cascade system also enabled the production of the ethylated SLC39A5 peptide. In the presence of L‐methionine and ATP, full SLC39A5 methylation was observed within 30 min. L‐Selenoethionine led to slower ethylation, reaching completion after 5 h, however, reaction with L‐ethionine required overnight incubation to achieve full conversion (Figures 4j–m, and S16). The SLC39A5‐His375et product was confirmed by MS/MS (Figure S15).

### 2.4 Computational Analyses of Histidine Ethylation by SETD3

**FIGURE 5:**
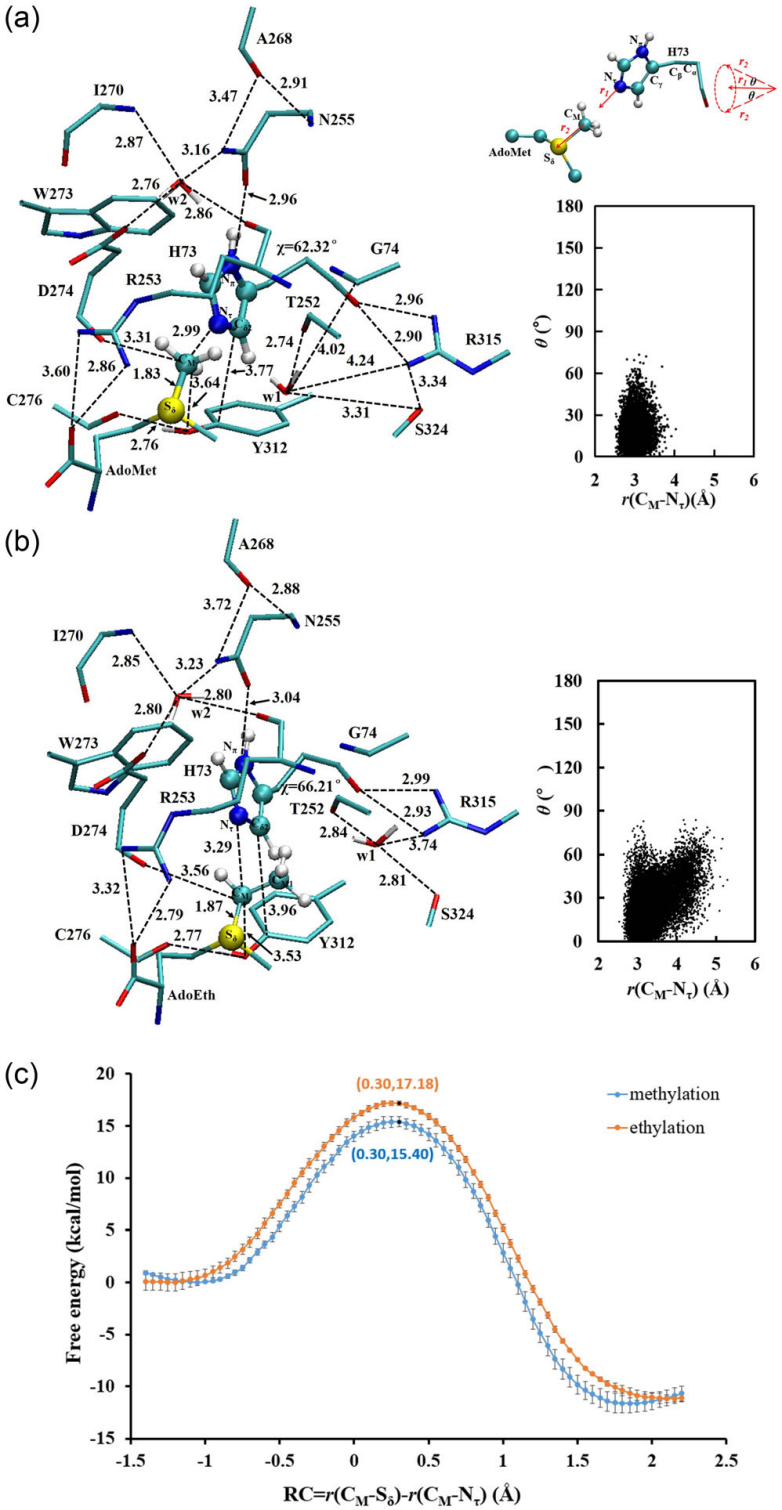
QM/MM MD and PMF results of SETD3. (a) The average active‐site structure of the reactant complex of SETD3 for methylation in the presence of AdoMet, along with *r*(C_M_‐N_τ_)‐*θ* distribution map, plotted based on the data from QM/MM MD simulations [20]. *θ* is the angle made by *r*_1_ and *r*_2_ as defined in this figure. The *r*_1_ direction approximates the lone‐pair electron direction of N_τ/π_, and *r*_2_ is a vector pointing from C_M_ to S_δ_. SETD3 is shown in sticks, and the S_δ_‐CH_3_ group from AdoMet and the imidazole ring of His73 are in ball and sticks. Some average distances from the simulations are also given (in angstroms) along with the average value of χ(N_τ_−C_β_−C_γ_−C_α_). (b) The average active‐site structure of the reactant complex of SETD3 for ethylation in the presence of AdoEth, along with *r*(C_M_‐N_τ_)‐*θ* distribution map obtained from QM/MM MD simulations. SETD3 is shown in sticks, and the S_δ_‐CH_2_‐CH_3_ group from AdoEth and the imidazole ring of His73 are in ball and sticks. (c) PMF free energy profiles for methylation and ethylation of His73 by SETD3.

To explore the molecular basis for histidine ethylation by SETD3 and METTL9 and to understand the structural origin of the lower catalytic efficiency of ethylation compared to methylation observed experimentally, we carried out a comparative QM/MM study and computational analyses for histidine methylation and ethylation reactions catalyzed by SETD3 and METTL9. The histidine methylation by SETD3, based on QM/MM simulations, has been investigated in earlier studies [20, 48]. The average active‐site structures of the reactant complexes of SETD3 for His73 methylation and ethylation are shown in Figure 5a,b, respectively. The imidazole ring of His73 has similar orientations; it is almost perpendicular to the aromatic ring of Tyr312 (as also observed in the crystal structure of the product complex containing His73me) [19, 49]. There are nevertheless, some important structural differences that may affect the catalytic efficiency. For instance, the interaction between the transferable methyl group of AdoMet and the carbonyl O atom of Asp274 seems to be weakened when AdoMet was replaced by AdoEth, with the average distance changing from 3.3 Å in Figure 5a to 3.6 Å in Figure 5b. More importantly, the distance between the transferable methyl group of AdoMet and the target N_τ_ atom of His73 is 3.0 Å for methylation, while the corresponding distance involving AdoEth is 3.3 Å. Consistent with the results based on the average structures (Figure S17), the distribution map of Figure 5a shows that the structures of the reactant complex for the methyl transfer generally have relatively short *r*(C_M_−N_τ_) distances during the molecular dynamics (MD) simulations (i.e., mainly between 2.5 and 3.5 Å). By contrast, the *r*(C_M_−N_τ_) distances for the reaction complex for ethylation can reach as much as ~5 Å (Figure 5b). The population of the structures with relatively large values of *θ* angle (e.g., 30º–70º) is significantly higher in Figure 5b compared to that in Figure 5a. The results indicate that the alignment of the lone pair of electrons on N_τ_ with the transferable ethyl group for ethylation of His73 (Figure 5b) is much worse compared to the alignment of the lone pair of electrons with the transferable methyl group for methylation (Figure 5a). The decrease of the population of the reactive near attack conformations for ethylation may contribute, at least in part, to the higher free energy barrier obtained for ethylation (17.2 kcal mol^−1^) than that for methylation (15.4 kcal mol^−1^) (Figure 5c). The QM/MM simulation results are consistent with the enzyme kinetics data in Table 1, which show that the *k*_cat_ value decreases significantly in going from methylation to ethylation, suggesting that the catalytic efficiency of ethylation by SETD3 is significantly lower than that of methylation.

It is of interest to discuss our results in connection with some of the previous results for S_*N*_2 reactions under nonenzymatic environments, which have been subjects of extensive experimental and theoretical investigations [50]. It has been demonstrated that in nonenzymatic environments, alkyl substituents to the central electrophilic carbon (e.g., from CH_3_X to C_2_H_5_X. Here X is the leaving group/anion) generally slow down S_*N*_2 reactivity with the rate decreasing (the barrier at TS increasing) in the order of CH_3_X > C_2_H_5_X > n‐C_3_H_7_X > n‐C_4_H_9_X > i‐C_3_H_7_X > t‐C_4_H_9_X. The increase of free energy barrier (i.e., 1.8 kcal mol^−1^) obtained here seems to be consistent with previous results for other S_*N*_2 reactions under nonenzymatic environments [50]. For instance, it was shown that the difference in the free energy barriers for the S_*N*_2 reactions of chloride with methyl and ethyl chlorides obtained from high‐level QM calculations is 1.9 kcal mol^−1^ in the gas phase; the differences are about 3.1–3.4 kcal mol^−1^ in different solutions [51]. It is widely believed that a main reason for the decrease of the rate of the reactions and increase of the corresponding barriers at TS is due to the steric hindrance in the case involving a relatively bulky ethyl group compared to a methyl group that prevents the efficient backside attack by a nucleophile. This seems to be the case, at least in part, for the SETD3 and METTL9 (see below) catalyzed reactions as well, as indicated by the increase of the *r*(C_M_−N_τ_) distances and change of the values of the *θ* angle in going from methylation to ethylation.

To help understand the potential origin of the loss of the activity because of the R253G mutation, we performed the simulations for βA‐His73 methylation by SETD3 R253G. It was found that the free energy barrier increases by about 3 kcal mol^−1^, due in part to the loss of the population of near attack conformation in the reaction state (Figure S18). As reported, it was observed that the G74S mutation of βA peptide led to ~50% reduction in k_cat_ for SETD3 [22]. We have also performed the QM/MM MD simulations for the reaction complex containing the βA‐G74S substituted peptide and found that, as a result of the G74S mutation, the formation of near attack conformations for methylation by SETD3 is significantly compromised, along with a rotation of the His73 ring from χ(N_π_−C_β_−C_γ_−C_α_) = 62º to 72.6º (Figure S19). There is also an increase in the free energy barrier for the mutated substrate (Figure S19), consistent with experimental observation.

### 2.5 Computational Analyses of Histidine Ethylation by METTL9

**FIGURE 6:**
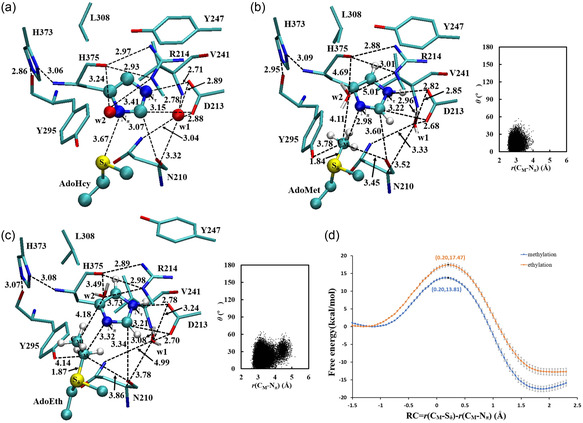
QM/MM MD and PMF results of METTL9. (a) The crystal structure (PDB ID: 7YF2) of METTL9 in complex with unmethylated SLC39A5 peptide and AdoHcy. (b) The average active‐site structure of the reactant complex of METTL9 for methylation in the presence of AdoMet, along with *r*(C_M_‐N_π_)‐*θ* distributions obtained from QM/MM MD simulations. METTL9 is shown in sticks, and the S_δ_‐CH_3_ group from AdoMet and the imidazole ring of His375 are in ball and sticks. (c) The average active‐site structure of the reactant complex of METTL9 for ethylation in the presence of AdoEth, along with *r*(C_M_‐N_π_)‐*θ* distributions obtained from QM/MM MD simulations. METTL9 is shown in sticks, and the S_δ_‐CH_2_‐CH_3_ group from AdoEth and the imidazole ring of His375 are in ball and sticks. (d) PMF free energy profiles for methylation and ethylation of SLC39A5‐His375 catalyzed by METTL9.

The comparison of the crystal structure of METTL9 (Figure 6a) [6] and the average active‐site structure of the reactant complex for methylation by METTL9 containing AdoMet and SLC39A5‐His375 substrate (Figure 6b) is shown in Figure 6. The key hydrogen bonding interactions remain basically the same in the crystal structure and reactant complex from the simulations. For instance, the interaction between the guanidine group of Arg214 and the carbonyl O atom of His375 is present in the average structure from the simulations, with the average distances of 2.9 and 3.0 Å, respectively, that are almost the same as those in the crystal structure. The N‐H···O and C–H···O hydrogen bonding interactions formed between the imidazole ring of SLC39A5‐His375 and the carboxylate group of Asp213 are also well maintained in the average structure of the reaction complex for methylation compared to those in the crystal structure. Some of these interactions are expected to contribute to the binding of the substrate and generate the near attack conformation for subsequent N_π_‐specific methylation. For instance, it has been demonstrated that the R214A variant abolished the binding to SLC39A5, suggesting the importance of this residue for productive catalysis [6]. In addition, water molecule w1 occupies almost the same position in the simulations as that observed in the crystal structure. It is worth noting that there are some differences in Figure 6a,b due in part to the lack of the transferable methyl group in the crystal structure (which contains AdoHcy instead). The transferable methyl group of AdoMet forms C–H···O hydrogen bonds with the phenolic hydroxyl group of Tyr295, the carbonyl O atom of Asn210, and the water molecule w2, with the average distances of 3.78, 3.45, and 4.11 Å, respectively (Figure 6b). The existence of the positively charged AdoMet in the reaction complex is expected to make some modification of the crystal structure.

The average active‐site structure of the reactant complex of METTL9 for AdoEth‐mediated ethylation of SLC39A5‐His375 obtained from QM/MM MD simulations is shown in Figure 6c. Comparison of the structures for methylation and ethylation shows that the key hydrogen bonding interactions involving Arg214 and Asp213 in the complex for ethylation (Figure 6c) are rather similar to those for methylation (Figure 6b). However, the hydrogen bonding interactions involving w1 in the complex for ethylation seem to be weakened compared to those for methylation. The C–H···O hydrogen bonding interactions involving the transferable methyl group with Tyr295 and Asn210 are also weakened based on the simulations, with the average distances changing from 3.78 and 3.45 Å for methylation to 4.14 and 3.86 Å for ethylation (Figure S17). Perhaps the most important change for the reaction complex in going from methylation to ethylation is the significant reduction of the population of the near‐attack conformations with good alignment of the lone pair of electrons with the transferable group. Indeed, as can be seen from the *r*(C_M_‐N_π_)‐*θ* distribution map in Figure 6c, the *r*(C_M_−N_π_) distances for the reaction complex for ethylation can reach as much as ~5 Å, while they are mainly in the range of 2.5–3.5 Å for methylation (Figure 6b). The free energy profiles for the methylation and ethylation reactions of SLC39A5‐His375 catalyzed by METTL9 are shown in Figure 6d. The free energy barrier of methylation (13.8 kcal mol^−1^) is lower than that of ethylation (17.5 kcal mol^−1^) by 3.7 kcal mol^−1^, demonstrating that methylation is far more efficient compared to ethylation in METTL9, consistent with our experimental observations.

## 3 Conclusions

Methylation of histidine residues in proteins has recently gained an interest in the chemical and biochemical community due to the discovery of the first histidine methyltransferases with roles in human biomedicine. Common for all methyltransferases is the use of AdoMet as the universal cosubstrate in the methylation reaction, and analogs of AdoMet have previously been shown to allow for the introduction of functional groups other than methyl onto protein, DNA, and RNA targets. Here, we have described the ability of recently characterized histidine methyltransferases SETD3 and METTL9 to catalyze methylation and most related ethylation reaction of histidine residues using individual and one‐pot cascade reactions. We showed that SETD3 can efficiently catalyze ethylation of βA‐His73 in the presence of the AdoSeEth cosubstrate, while AdoEth appears to be a relatively poorer cosubstrate. Kinetic evaluations showed that ethylation results in a significant loss of efficiency in comparison to methylation, but activity could be recovered by the replacement of the sulfonium moiety with a more reactive selenonium group. In contrast, METTL9 could not efficiently catalyze ethylation of SLC39A5‐His375 in the presence of AdoEth and AdoSeEth under standard conditions; however, with the increase in the concentration of METTL9, a higher degree of ethylation could be observed. However, the MAT‐SETD3 cascade system allows an efficient (m)ethylation of the βA peptide using L‐methionine, L‐ethionine, and L‐selenoethionine with ATP. Similarly, the MAT‐METTL9 cascade system allowed (m)ethylation of the SLC39A5 peptide in the presence of L‐methionine, L‐ethionine, and L‐selenoethionine with ATP; however, longer incubations were required to achieve full conversions. Our QM/MM MD and free‐energy simulations provided energetic, structural, and dynamic interpretations for the experimental observations concerning why the catalytic efficiencies for ethylation of βA‐His73 and SLC39A5‐His375 are significantly lower than those of methylation. Taken together, our integrated experimental and computational studies provide an important biomolecular insight into the cosubstrate tolerance by histidine methyltransferases, knowledge useful for functional assignment and development of chemical probes targeting histidine methyltransferases. Overall, the work expands the application of chemical and biochemical methods for the site‐directed alkylation of histidine residues in peptides and proteins.

## 4 Experimental

### 4.1 Peptide Synthesis and Purification

The β‐actin peptide (residues 66–81, TLKYPIEHGIVTNWDD) and SLC39A5 peptide (residues 369–380, GHQGHSHGHQGG) were chain assembled on Rink amide resin using microwave‐assisted SPPS on a Liberty Blue peptide synthesizer (CEM corporation, Matthews, NC, USA). All amino acid couplings were carried out with the equivalent ratio of [5][5]:[7.5] of [Fmoc‐protected amino acid]:[DIC]:[Oxyma Pure] at 75°C for 2 min or at 50°C for 4 min for histidine. The peptide proceeded to standard cleavage from resin using 0.5% TIPS, 0.5% H_2_O in conc. TFA. TFA was blown off using N_2_, and the resultant residue was suspended in cold Et_2_O. After suspension, it was subjected to centrifugation for 5 min at 5000 rpm in an Eppendorf 5804R centrifuge (Eppendorf, Hamburg, Germany), after which the supernatant was decanted into the waste. The remaining white to yellow solid was washed twice by cold Et_2_O and subjected to centrifugation, after which the crude peptide was dissolved in a mixture of ACN in H_2_O and purified using preparative reverse‐phase HPLC (RP‐HPLC) using a gradient of buffer A and buffer B from 20% B to 70% over 40 min at 4 mL min^−1^ using a Gemini 10 µm NX‐C18 110 Å LC column (Phenomenex, Torrance, CA, USA). The SLC39A5 peptide (residues 369–380, GHQGHSHGHQGG) was chain assembled on Rink amide resin via automated SPPS Purepep Chorus peptide synthesizer (Gyros Protein Technologies). All amino acid couplings were carried out with the equivalent ratio of [3][3]:[5]: of [Fmoc‐protected amino acid]:[HATU]:[DIPEA] at 50°C for 15 min and deprotection for 7 min was carried out after each coupling with 20% piperidine in DMF. The peptide was cleaved from resin using a cleavage cocktail comprising 2.5% TIPS, 2.5% MQ H_2_O, and 95% TFA after 3 h. TFA was blown off using N_2_ and the obtained residue was suspended in cold Et_2_O. After suspension, it was subjected to centrifugation for 5 min at 5000 rpm in an Eppendorf 5804R centrifuge (Eppendorf, Hamburg, Germany), after which the supernatant was decanted into the waste. The remaining pale‐yellow solid was washed twice with cold Et_2_O, centrifuged, and dried by blowing N_2_. The resultant dried crude peptide was dissolved in a mixture of ACN in H_2_O (1:1) and purified using preparative reverse‐phase HPLC (RP‐HPLC) using an isocratic gradient with buffer 20% ACN over 32 min at 3 mL min^−1^ using a Gemini 10 µm NX‐C18 110 Å LC column (Phenomenex, Torrance, CA, USA). Analytical RP‐HPLC for both peptides was carried out on a Gemini 5 µm C18 110 Å LC column (Phenomenex) at a flow rate of 1 mL min^−1^. Analytical injections were monitored at 215 nm.

### 4.2 Enzyme Expression and Purification

The bacterial expression plasmid for human SETD3 fused to an N‐terminal His_6_‐tag was constructed employing a pCOLD I vector (Takara Bio, Kusatsu, Japan) as described previously [4]. The recombinant enzyme was produced and purified by a slightly modified procedure also described by Kwiatkowski et al. [4] Briefly, nine hundred mL of LB broth (with 100 mg mL^−1^ ampicillin) was inoculated with 50 mL of an overnight pre‐culture of *E. coli* BL21(DE3) harboring the plasmid and incubated at 37°C and 200 rpm until an OD_600_ of 0.6 was reached. The expression of the enzyme was induced by placing the culture on ice for 20 min (cold‐shock) and the addition of IPTG to a final concentration of 0.3 mM. Cells were incubated for 16 h at 13°C, 200 rpm, and harvested by centrifugation (5000 × g for 10 min). The pelleted bacteria were then resuspended in 50 mL lysis buffer consisting of 25 mM HEPES pH 7.5, 300 mM NaCl, 10 mM KCl, 1 mM DTT, 2 mM MgCl_2_, 1 mM PMSF, 0.25 mg mL^−1^ hen egg white lysozyme (BioShop, Canada), and 1100 U Viscolase (A and A Biotechnology, Poland). The cells were lysed by freezing in liquid nitrogen and, after thawing and vortexing, the extracts were centrifuged at 4°C (20000 × g for 20 min). For the purification of SETD3, the supernatant of *E. coli* lysate (50 mL) was diluted with 100 mL of buffer A (50 mM HEPES, pH 7.5, 400 mM NaCl, 10 mM KCl, 30 mM imidazole, 1 mM DTT) and applied onto a HisTrap FF Crude column (5 mL) equilibrated with the same buffer. The column was then washed with 30 mL buffer A, and the retained proteins were eluted with a stepwise gradient of imidazole (25 mL of 60 mM, 24 mL of 150 mM, and 23 mL of 300 mM) in buffer A. The recombinant protein was present in both 150 and 300 mM imidazole fractions, but only the SETD3 protein eluted at the highest concentration of imidazole, exhibiting >95% purity as confirmed by SDS‐PAGE, and further processed. The enzyme preparation was desalted onto PD‐10 columns equilibrated with 20 mM Tris‐HCl, pH 7.2, 50 mM KCl, 1 mM DTT, and 6% sucrose. The yield of the recombinant enzyme was about 8.0 mg of homogenous SETD3 per 500 ml of culture. The purified enzyme was aliquoted and stored at −70°C.

Mutated forms of the SETD3 enzyme (R253A and R253G) were generated by site‐directed mutagenesis using a QuikChange II XL kit (Agilent, USA), with the pCOLD I – SETD3 plasmid as the template and mutagenic primers listed in Table S1. Both mutated forms of SETD3 were produced in *E. coli* BL21(DE3) and purified using HisTrap FF Crude column (5 mL) as described for the WT enzymes (Figure S20).

The coding sequence for the human METTL9 gene (NM_016025.5) was obtained from the DNASU Plasmid Repository (HsCD00936521). The open reading frame, flanked by NdeI and XhoI restriction sites, was amplified via PCR and cloned into the pCOLD I vector (Takara Bio, Japan). This vector facilitates the production of recombinant proteins containing an N‐terminal His_6_‐tag in *E. coli*. The integrity of the resulting construct was confirmed by DNA sequencing (Eurofins Genomics, Germany). Recombinant METTL9 was overexpressed in *E. coli* BL21(DE3) cells (Agilent, USA) cultured in 3 liters of LB broth supplemented with 100 μg mL^−1^ ampicillin. Following induction with 50 µM IPTG, the culture was incubated for 24 h at 18°C with shaking at 175 rpm. Cells were harvested by centrifugation (4,000 × g for 10 min), and the pellet was resuspended in 150 mL of ice‐cold lysis buffer (50 mM HEPES, pH 8.0, 300 mM NaCl, 20 mM KCl, 1 mM DTT) supplemented with 2 μg  mL^−1^ leupeptin, 2 μg mL^−1^ antipain, 1% Triton X‐100, 0.25 mg mL^−1^ hen egg white lysozyme, 1 mM PMSF, and 3000 U Viscolase. The lysate was cleared by centrifugation at 20,000 × g for 30 min at 4°C. The clarified supernatant was diluted threefold with buffer A (50 mM HEPES, pH 8.0, 500 mM NaCl, 10 mM KCl, 1 mM DTT, 30 mM imidazole) and loaded onto a 5 mL HisTrap FF Crude column pre‐equilibrated with the same buffer. The protein was eluted using a stepwise imidazole gradient (60, 150, and 300 mM) in buffer A, with the highest concentration of METTL9 recovered in the 300 mM fraction. This fraction underwent sequential dialysis against a storage buffer (20 mM Tris‐HCl, pH 7.5, 150 mM NaCl, 10 mM KCl, 1 mM DTT, 10% sucrose) consisting of an overnight exchange followed by two 3 h exchanges at 6°C. The purification yielded approximately 1 mg of protein per 3 liters of culture with >90% purity (as determined by SDS‐PAGE). Finally, the protein was concentrated to ~9 μM using an Amicon Ultra Centrifugal Filter, aliquoted, and stored at −80°C.

PC‐MjMAT was expressed as described previously [37, 44]. Electrocompetent *E. coli* BL21(DE3) cells were transformed with the corresponding plasmid. Main cultures were inoculated with 2% of the pre‐culture. Cells were grown in LB medium containing 100 µg mL^−1^ kanamycin at 37°C until OD600 reached 0.6. The culture was induced using 0.5 mM S4 isopropyl‐β‐D‐1‐thiogalactopyranoside (IPTG) and incubated at 37°C for 3 h with 180 rpm. After production, cells were centrifuged at 3500 g for 15 min and stored at −80°C. Proteins were purified using their His‐tag by immobilized metal affinity chromatography (IMAC). Cell pellets were resuspended in lysis buffer (50 mM Tris‐HCl, pH 8.0, 300 mM NaCl, 10 mM imidazole) and sonicated for 3 x 3 min at an amplitude of 30 % using a Sonopuls HD 4100 (Bandelin, Berlin). The cell lysate was centrifuged at 21,000 g for 30 min, and the supernatant was filtered through a 0.2 µM filter. The purification was performed using the ÄKTA start. A HisTrap FF column (5 mL) was equilibrated with lysis buffer, the sample was aspirated, and proteins were eluted by an increasing gradient of imidazole (elution buffer: 50 mM Tris‐HCl, pH 8.0, 300 mM NaCl, 500 mM imidazole). Fractions containing the desired protein were pooled. Concentration and buffer exchange to storage buffer (25 mM Tris‐HCl, pH 8.0, 80 mM KCl, 10% glycerol) was performed using Amicon Ultra‐15 centrifugal units (30 kDa cut‐off). Concentrated proteins were aliquoted, flash frozen in liquid nitrogen, and stored at −80°C.

### 4.3 Enzyme Assays

SETD3's enzymatic activity toward βA peptides was measured at different time points under standard conditions (1 µM SETD3 enzyme, 10 µM peptide, 100 µM AdoMet/AdoEth/AdoSeEth) or with elevated concentration of SETD3 (5 μM) in the reaction buffer at pH 9.0 (25 mM HEPES, 20 mM NaCl). Ultimately, SETD3 (5 μM) was preincubated with AdoMet, AdoEth, and AdoSeEth (100 μM) for 5 min after which the reaction was carried out normally. The reactions were carried out in a final volume of 50 µL by incubation by shaking in a Thermomixer C (Eppendorf) at 750 rpm, at 37°C. All reactions were quenched by the addition of 10% TFA in MilliQ water (v/v), aliquoted and mixed 1:1 with α‐cyano‐4‐hydroxycinnamic acid (CHCCA) matrix dissolved in a mixture of H_2_O and ACN (1:1, v/v), and loaded onto an MTP 384 polished steel target to be analyzed by an UltrafleXtreme‐II tandem mass spectrometer (Bruker).

METTL9 enzymatic activity toward SLC39A5 peptide was evaluated at different time points under standard conditions (1 µM METTL9 enzyme, 10 µM peptide, 100 µM AdoMet/AdoEth/AdoSeEth) and with elevated concentration of METTL9 (5 μM) in the reaction buffers at pH 7.5 (20 mM Tris‐HCl, 50 mM NaCl, 1 mM MgCl_2_, and 1 mM DTT) and pH 9.0 (25 mM Tris‐HCl, 20 mM NaCl). METTL9 (1 μM or 5 μM) was preincubated with AdoMet, AdoEth, and AdoSeEth (100 μM) for 5 min in buffer. Peptide SLC39A5 (10 μM) was added afterwards. The reactions (final volume 20 µL) were carried out by incubation by shaking in a Thermomixer C (Eppendorf) at 750 rpm, at 37°C. All reactions were quenched after 1 and 3 h by the addition of 10% TFA in MilliQ water (v/v), aliquoted and mixed 1:1 with α‐cyano‐4‐hydroxycinnamic acid (CHCCA) matrix dissolved in a mixture of H_2_O and ACN (1:1, v/v), and loaded onto an MTP 384 polished steel target to be analyzed by an UltrafleXtreme‐II tandem mass spectrometer (Bruker).

MTA assays with L‐methionine, L‐ethionine, and L‐selenoethionine with PC‐MjMAT were performed under the same conditions: 5 mM L‐methionine, L‐ethionine, and L‐selenoethionine were incubated with 5 mM ATP and 100 μM PC‐MjMAT in a total volume of 30 μL. 10 µL are used as a 0 h sample, 10 µL are incubated at 37°C for 1 h, and 10 µL are incubated at 65°C for 1 h. Reaction buffer (1×) consisted of 50 mM HEPES, 10 mM MgCl_2_, 5 mM KCl (pH 7.4). Samples were taken at the indicated time points, acidified by the addition of 10% (v/v) of 1 M HClO_4_, and centrifuged for 15 min at 21 130 × g for 2 times; the supernatant was filtered with a syringe‐driven filter, and 3 μL of supernatant was directly analyzed via HPLC.

The enzymatic methylation and ethylation assays for SETD3 and METTL9 were carried out as MAT/MTase cascade reactions, and the enzymatic activities were measured at different time points. The MAT reaction was identical for both methyltransferases and contained 1 mM ATP, 2 mM L‐methionine, L‐ethionine or L‐Se‐ethionine, and 25 µM PC‐MjMAT in a pH 8.0 buffer (50 mM HEPES, 5 mM KCl, and 10 mM MgCl_2_). Additionally, 5 µM MTAN, which was produced and purified as previously described [44, 52], was included to degrade the byproduct S‐adenosylhomocysteine analogs. For SETD3 reactions, 5 µM SETD3 and 100 µM β‐actin peptide were used. For METTL9 reactions, 2 µM METTL9 and 20 µM SLC39A5 peptide substrate were employed. Reactions were incubated at 37°C with shaking at 750 rpm using a Thermomixer C (Eppendorf) for up to 24 h. All reactions were quenched by the addition of 10% TFA in MilliQ water (v/v), aliquoted and mixed 1:1 with α‐cyano‐4‐hydroxycinnamic acid (CHCCA) matrix dissolved in a mixture of H_2_O and ACN (1:1, v/v), and loaded onto an MTP 384 polished steel target to be analyzed by an UltrafleXtreme‐II tandem mass spectrometer (Bruker). The conversion percentages for enzymatic reactions were calculated by integrating the intensity of the observed MALDI‐TOF MS peaks for unalkylated substrates and alkylated products using flexAnalysis (Bruker).

### 4.4 Enzyme Kinetics Assays

βA peptide kinetic evaluation was carried out with a MALDI‐TOF MS assay under steady‐state conditions at pH 9. AdoMet, AdoEth, AdoSeEth (0–50 μM) and βA peptide (10 μM) were incubated, and reactions were started by the addition of SETD3 (250 nM, depending on the tested analog) in a final volume of 25 μL. The reactions were carried out by incubation by shaking in a Thermomixer C (Eppendorf) at 750 rpm, at 37°C. Reactions were quenched by the addition of 10% TFA in MQ after 20 min. All reactions were aliquoted, mixed 1:1 with α‐cyano‐4‐hydroxycinnamic acid (CHCCA) in a mixture of H_2_O and ACN (1:1, v/v) and loaded onto an MTP 384 polished steel target to be analyzed by a MALDI‐TOF MS. The amount of alkylated peptide was calculated by integration of the product peak area and divided by the amount of total (alkylated and unalkylated) peptide, taking into account all the ionic species. Kinetic values were extrapolated by fitting V_0_ values and cosubstrate concentrations to the Michealis–Menten equation using GraphPad Prism 5. Experiments were carried out in triplicate and final values are reported as value ± SE.

### 4.5 QM/MM Computations

QM/MM MD and free energy (PMF) simulations were performed for methylation and ethylation using the CHARMM program [53]. The –CH_2_–CH_2_–S^+^(Me/Eth)–CH_2_– part of AdoMet/AdoEth and His73 (SETD3)/His375 (METTL9) side chain was treated by QM, and the rest of the system by MM. The link‐atom approach [54] was applied to separate the QM and MM regions. A modified TIP3P water model [55] was employed for the solvent, and the stochastic boundary MD method [56] was used for the QM/MM MD and free energy (PMF) simulations with the hybrid QM/MM potential energy functions. The DFTB3 method [57, 58] implemented in CHARMM was used for the QM atoms, and the all‐hydrogen CHARMM potential function (PARAM27) [59] was used for the MM atoms. The structures of the reaction state for methylation and ethylation were generated based on the crystal structures of the SETD3 complex containing His73 and sinefungin (SFG) (PDB ID: 6OX0) [49] and the METTL9 complex containing His375 and AdoHcy (PDB ID: 7YF2) [6]. SFG in 6OX0 and AdoHcy in 7YF2 were changed to AdoMet/AdoEth manually. The imidazole ring of His73 was generated as the N_π_‐H π tautomer with N_τ_ unprotonated, and the imidazole ring of His375 was generated as the N_τ_‐H τ tautomer with N_π_ unprotonated. The QM/MM MD simulations were performed with the hybrid QM/MM potential energy functions for these two enzymes. To make sure that AdoEth adopts a meaningful configuration, several different conformations of the ethyl group of AdoEth were used as the starting conformations. All the QM/MM MD simulations with the different initial structures led to a similar conformation in each case within 100 ps of the simulations. The QM/MM MD simulations were then continued with the converged conformations for another 5 ns. The average structures were found to be essentially the same based on the results of 2 ns and 5 ns of the QM/MM MD simulations, suggesting that the structures for the reactant states are unlikely to change further with additional QM/MM MD simulations. The average structures and r(C_M_‐N_τ_)‐θ distribution maps were generated based on the data from the 2 ns QM/MM MD simulations; the results based on 5 ns for ethylation are given in the Supporting Information. Here, *θ* is the angle made by r_1_ and r_2_ as defined in Figure 5. The r_1_ direction approximates the lone‐pair electron direction of N_τ_/_π_, and r_2_ is a vector pointing from C_M_ to S_δ_. The umbrella sampling method [60] implemented in the CHARMM program, along with the Weighted Histogram Analysis Method (WHAM) [61], was applied to determine the change of the free energy (potential of mean force or PMF) as a function of the reaction coordinate for the methyl/ethyl transfer from AdoMet/AdoEth to His73 in SETD3 and His375 in METTL9, respectively. The reaction coordinates of methylation and ethylation were defined as a linear combination of r(C_M_‐N_τ/π_) and r(C_M_‐S_δ_) [R = r(C_M_‐S_δ_)–r(C_M_‐N_τ/π_)].

## Funding

This study was supported by the Independent Research Fund Denmark (Grant 10.46540/3105‐00324B), Opus‐14 grant from the National Science Centre, Poland (Grant 2017/27/B/NZ1/00161), the Natural Science Foundation of China (Grant 22177064) and the Natural Science Foundation of Shandong Province (Grant ZR2021MB050). A.R. thanks the DFG for funding (Grant RE2796/10−1) within the research unit FOR 5596 (Grant 510974120).

## Conflicts of Interest

The authors declare no conflicts of interest.

## Supporting information

Supplementary Material

## Data Availability

The data that support the findings of this study are available in the supplementary material of this article.
