# Supplementary material for: Histidine Ethylation by Histidine Methyltransferases SETD3 and METTL9
Source: Chembiochem. 2026 Jun 17;27(12):e70403. doi: 10.1002/cbic.70403 (PMC13274687; doi:10.1002/cbic.70403)
Supplement: Supplementary file 1 — Supplementary Material [file CBIC-27-e70403-s001.pdf]

Supporting Information

Histidine Ethylation by Histidine Methyltransferases SETD3 and METTL9

1. Experimental Section

Materials

All reagents were obtained from commercial sources and used without further purifications. Water was purified using a Millipore Milli-Q water purification system (Merck-Millipore, Burlington, MA, USA). For HPLC the following buffers were used: Buffer A: H_2O (0.1% TFA); Buffer B: ACN (0.1% TFA). Lyophilization of purified peptides was achieved using a VaCo 2 lyophilizer (Zirbus Technology GmbH, Bad Grund, Germany).

2. MALDI-TOF MS SETD3 methylation supporting figures

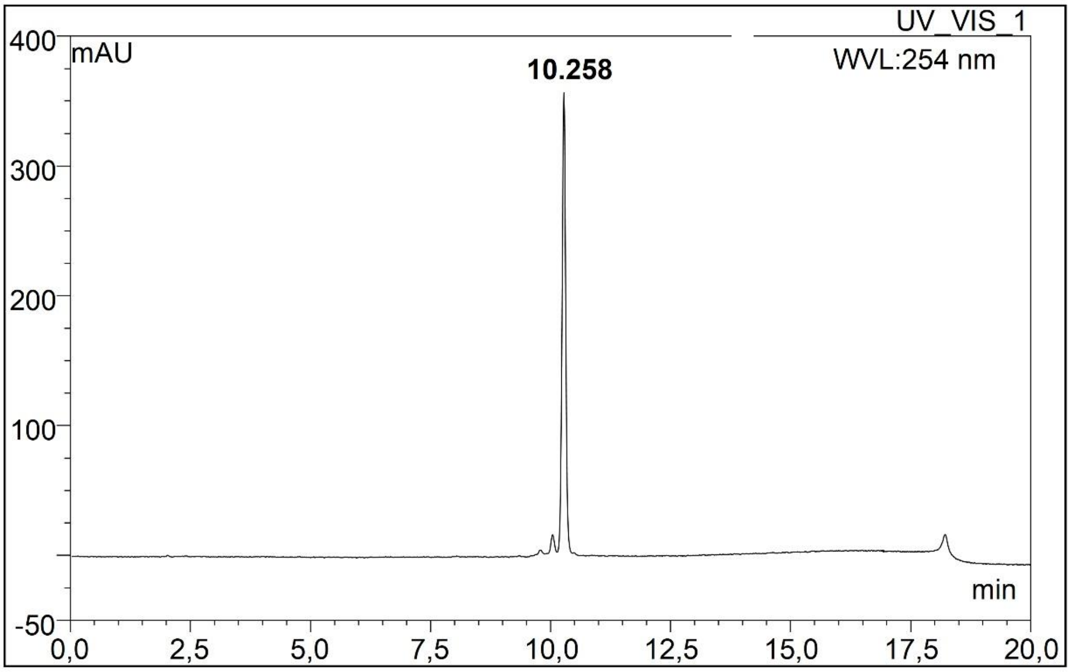

Figure S1. Analytical HPLC of the βA-His73 peptide after RP-HPLC purification.

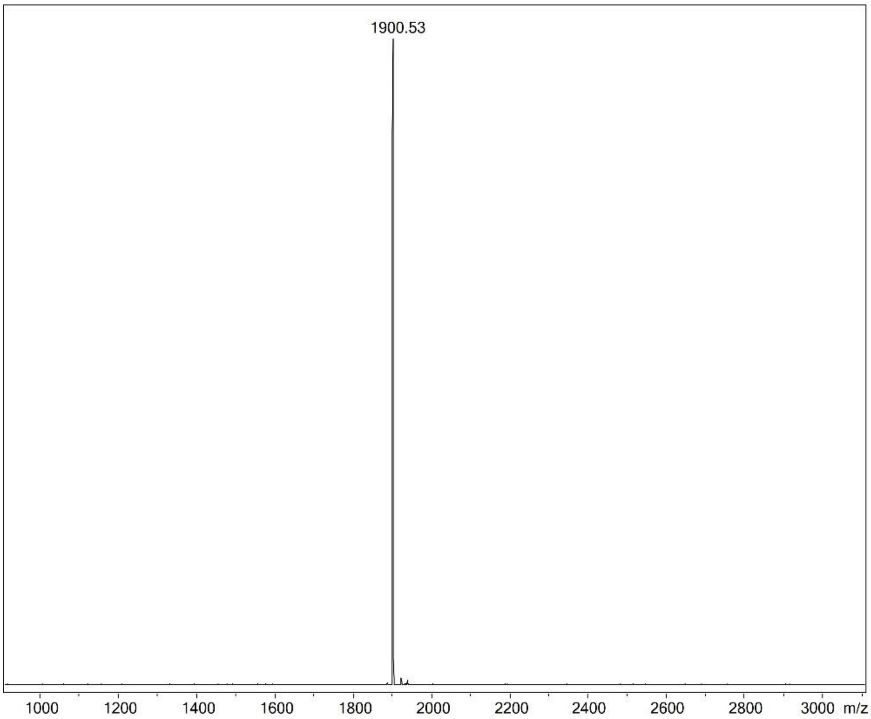

Figure S2. MALDI-TOF spectrum of the purified βA-His73 peptide.

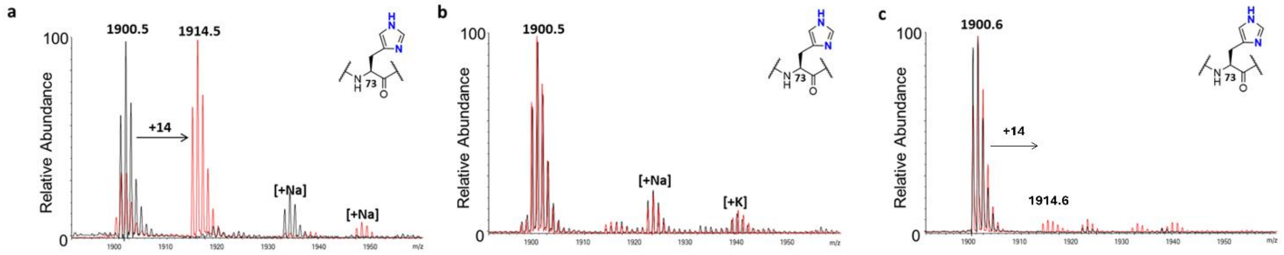

Figure S3. MALDI-TOF MS data showing reactions of a) βA-His73 peptide (10 μM), AdoMet (100 μM) and SETD3 (1 μM) and controls in absence of b) SETD3 and c) AdoMet after 3 hours at pH 9.0.

3. MALDI-TOF MS SETD3 histidine methylation and ethylation supporting figures

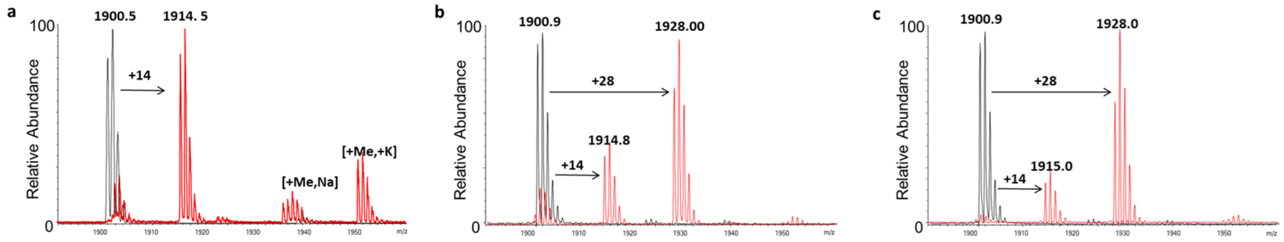

Figure S4. MALDI-TOF MS data showing alkylation of the $\beta A_{66\text{-}81}$ peptide (10 μM) in presence of SETD3 (5 μM) and AdoMet analogs (100 μM) after 3 hours reaction at pH 9.0. Control reactions without SETD3 present are shown in black, SETD3-catalyzed reactions are shown in red. a) AdoMet b) AdoEth, c) AdoSeEth.

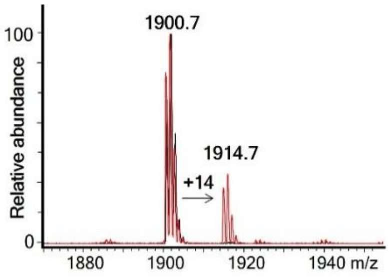

Figure S5. MALDI-TOF MS data showing methylation of the $\beta A_{66\text{-}81}$ peptide (10 μM) in presence of SETD3 (5 μM) after 3 hours at 37 °C at pH 9.0. Control reaction without SETD3 present is shown in black, SETD3-catalyzed reaction is shown in red. No AdoMet, AdoEth or AdoSeEth was added.

4. MALDI-TOF MS SETD3 mutants histidine methylation and ethylation supporting figures

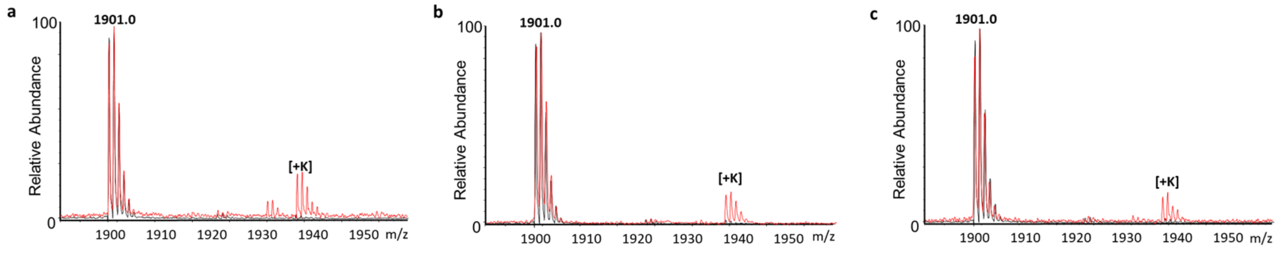

Figure S6. MALDI-TOF MS data showing alkylation of the $\beta A_{66\text{-}81}$ peptide (10 μM) in presence of SETD3 R253A (1 μM) and AdoMet analogs (100 μM) after 3 hours reaction at pH 9.0. Control reactions without SETD3 present are shown in black, SETD3-catalyzed reactions are shown in red. a) AdoMet b) AdoEth, c) AdoSeEth.

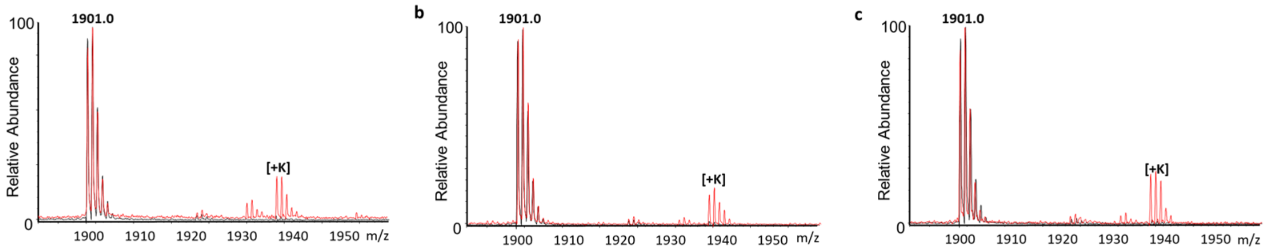

Figure S7. MALDI-TOF MS data showing alkylation of the βA$_{66\text{-}81}$ peptide (10 μM) in presence of SETD3 R253G (1 μM) and AdoMet analogs (100 μM) after 3 hours reaction at pH 9.0. Control reactions without SETD3 present are shown in black, SETD3-catalyzed reactions are shown in red. **a)** AdoMet **b)** AdoEth, **c)** AdoSeEth.

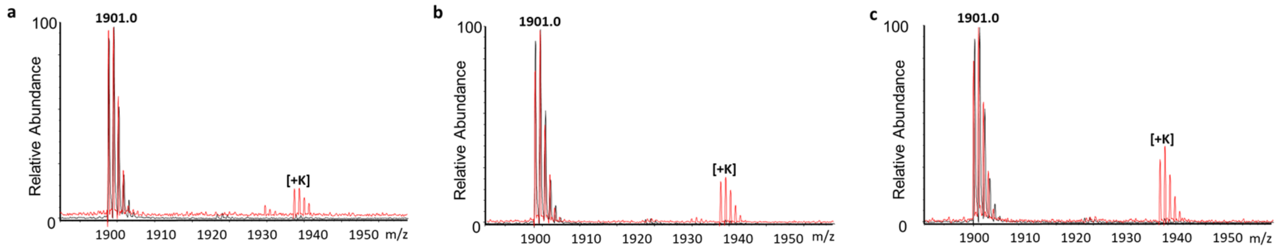

Figure S8. MALDI-TOF MS data showing alkylation of the βA$_{66\text{-}81}$ peptide (10 μM) in presence of SETD3 R253A (5 μM) and AdoMet analogs (100 μM) after 5 minutes preincubation and 3 hours reaction at pH 9.0. Control reactions without SETD3 present are shown in black, SETD3-catalyzed reactions are shown in red. **a)** AdoMet **b)** AdoEth, **c)** AdoSeEth.

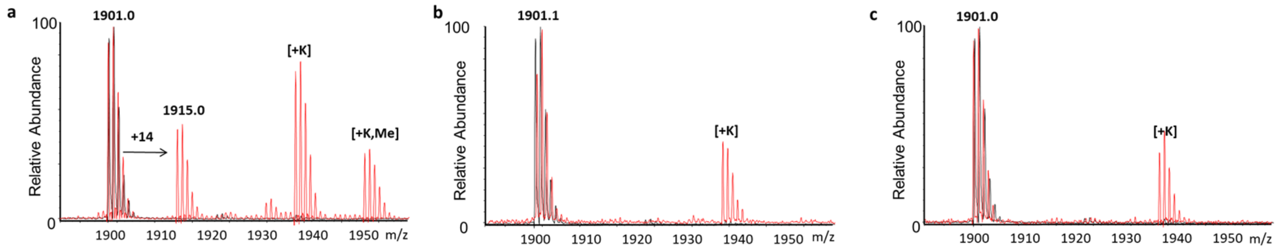

Figure S9. MALDI-TOF MS data showing alkylation of the βA$_{66\text{-}81}$ peptide (10 μM) in presence of SETD3 R253G (5 μM) and AdoMet analogs (100 μM) after 5 minutes preincubation and 3 hours reaction at pH 9.0. Control reactions without SETD3 present are shown in black, SETD3-catalyzed reactions are shown in red. **a)** AdoMet **b)** AdoEth, **c)** AdoSeEth.

5. MALDI-TOF MS METTL9 histidine methylation and ethylation supporting figures

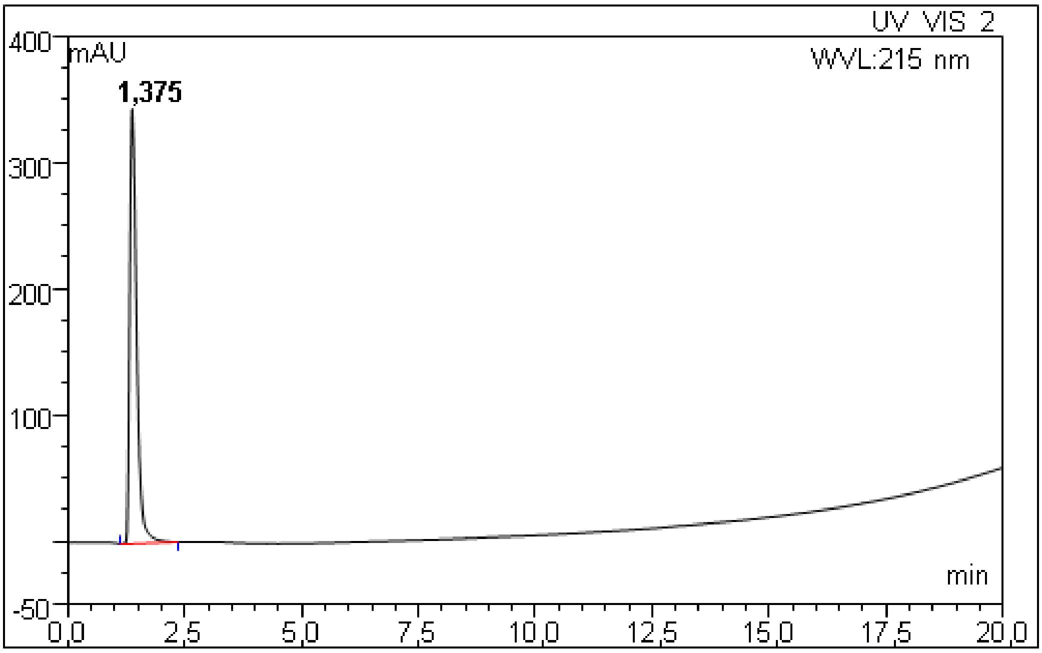

Figure S10. Analytical HPLC of the SLC39A5-His375 peptide after RP-HPLC purification.

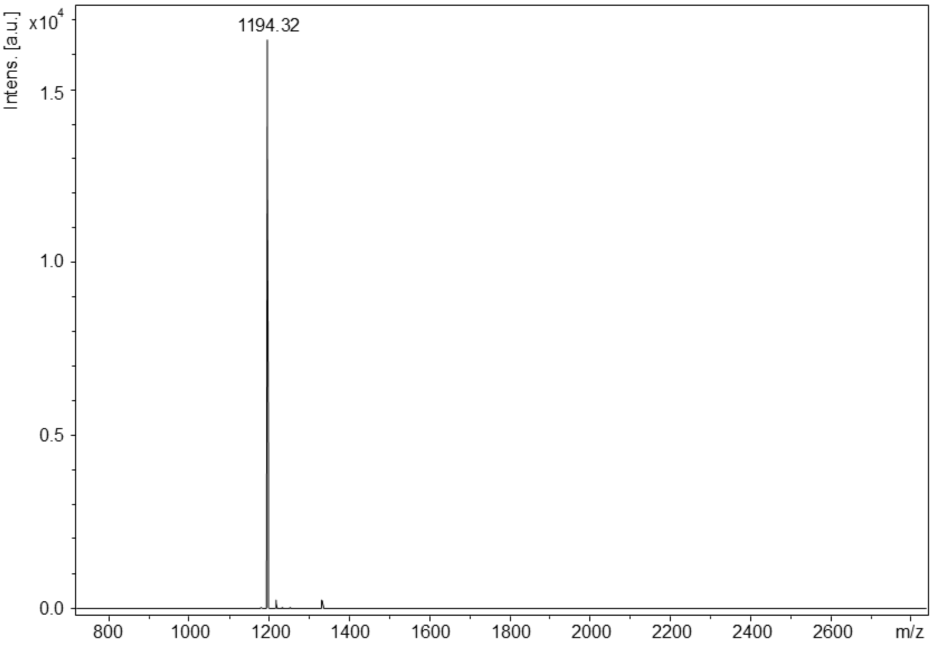

Figure S11. MALDI-TOF MS spectrum of the purified SLC39A5 peptide.

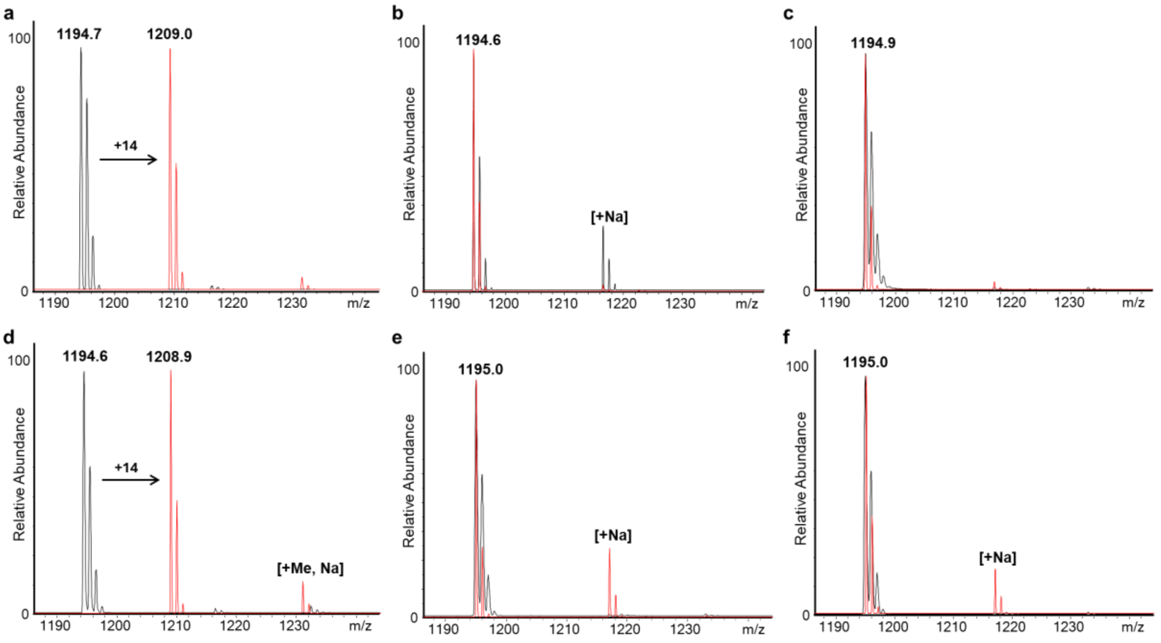

Figure S12. MALDI-TOF MS data showing alkylation of the $SLC39A5_{369-380}$ peptide (10 µM) in presence of METTL9 (1 µM) and SAM analogs (100 µM) after 5 minutes preincubation and 3 hours reaction. Control reactions without METTL9 present are shown in black, METTL9-catalyzed reactions are shown in red. a) AdoMet at pH 7.5 b) AdoEth at pH 7.5, c) AdoSeEth at pH 7.5, d) AdoMet at pH 9.0, e) AdoEth at pH 9.0, f) AdoSeEth at pH 9.0.

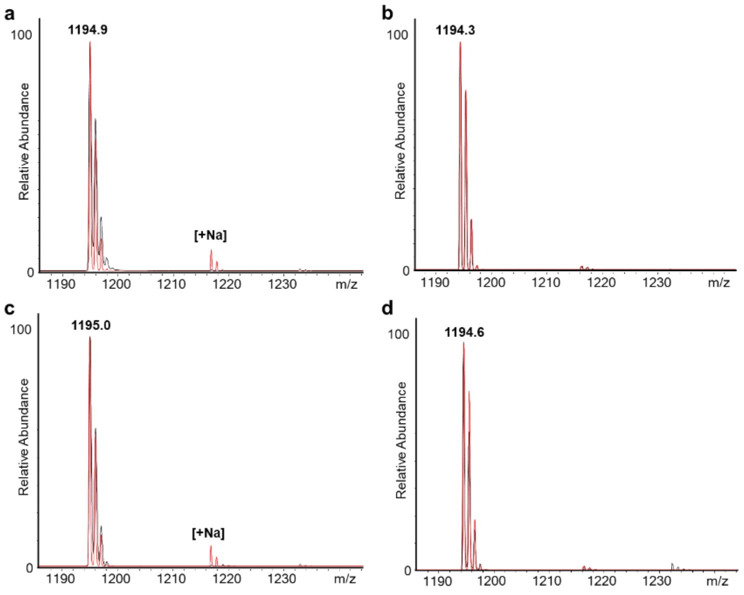

Figure S13. MALDI-TOF MS data showing negative control reactions of SLC39A5 peptides (10 µM) in the absence of (a) METTL9 at pH 7.5 (b) SAM at pH 7.5 (c) METTL9 at pH 9.0, (d) SAM at pH 9.0 after 3 hours.

6. Cascade reactions supporting figures

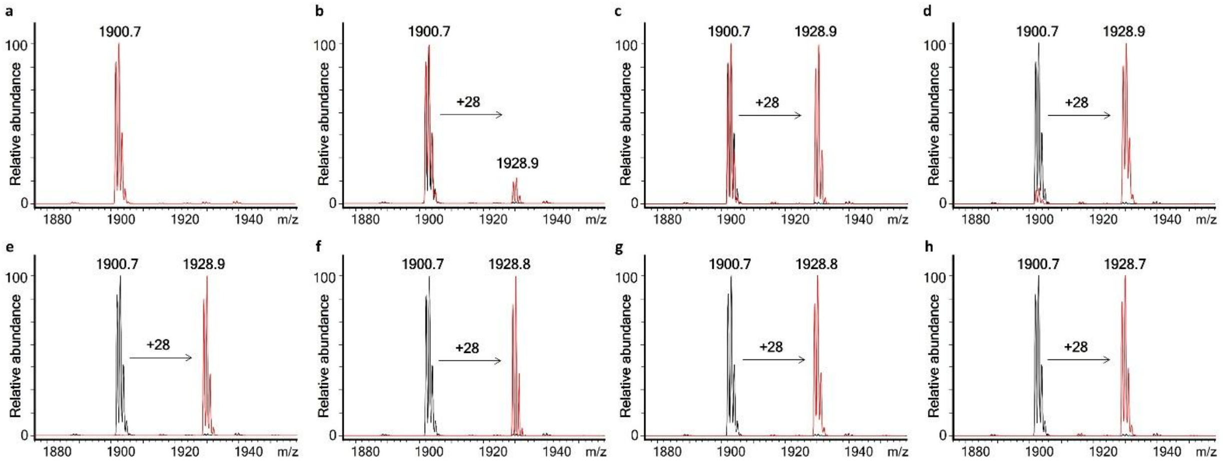

Figure S14. MALDI-TOF MS data for time-course of SETD3-catalyzed ethylation of the βA peptide in the presence of ATP and L-ethionine after a) 10 min, b) 30 min, c) 1 hour, d) 2 hours, e) 3 hours, f) 5 hours, g) 7 hours, h) 24 hours.

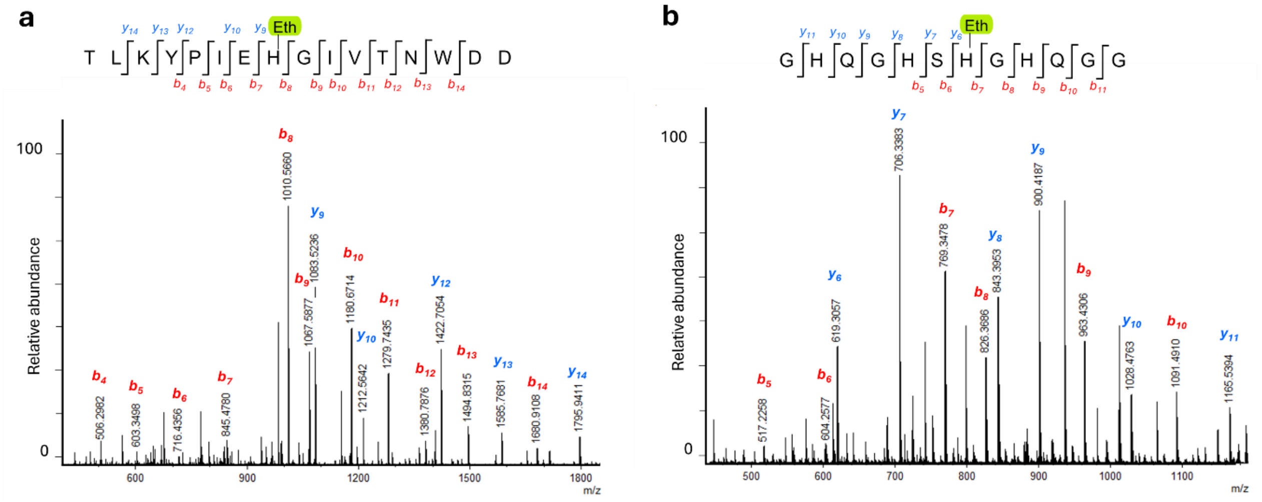

Figure S15. a) tims TOF flex MALDI-2 MSMS fragmentation data of a) Ethylated βA $_{66\text{-}81}$ produced by cascade reaction with PC-MjMAT and SETD3, b) Ethylated SLC39A5$_{369\text{-}380}$ produced by cascade reaction with PC-MjMAT and METTL9. b-ion series are shown in red and y-ion series are shown in blue.

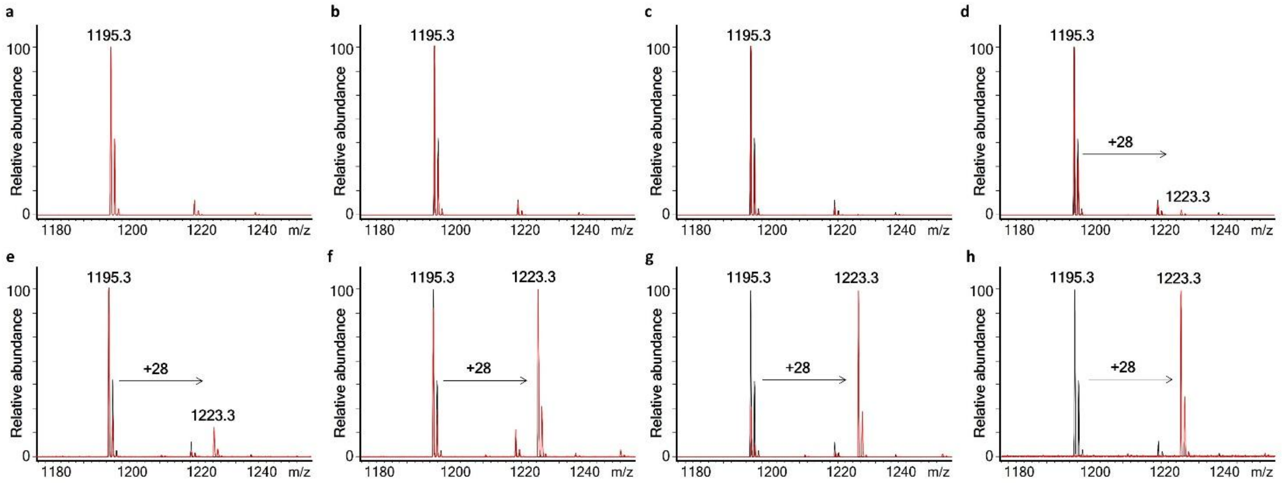

Figure S16. MALDI-TOF MS data for time-course of METTL9-catalyzed ethylation of the SLC39A5 peptide in the presence of ATP and L-ethionine after a) 10 min, b) 30 min, c) 1 hour, d) 2 hours, e) 3 hours, f) 5 hours, g) 7 hours, h) 24 hours.

7. QM/MM supporting figures

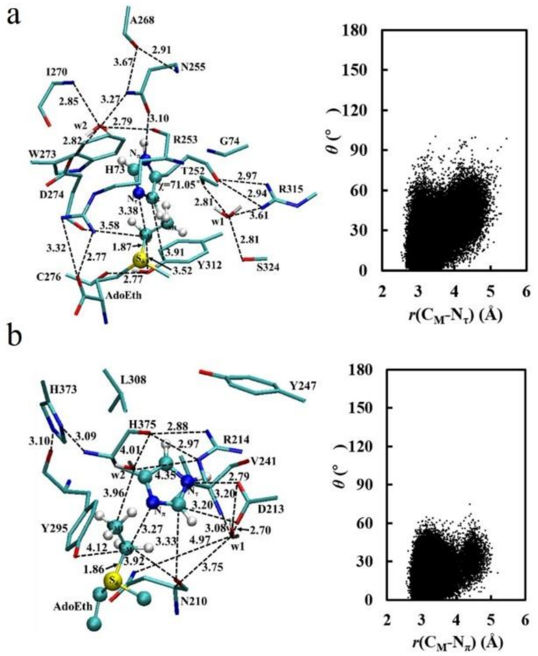

Figure S17. a) The average active-site structure of the reactant complex of SETD3 for ethylation in the presence of AdoEth along with r(C_M-N_τ)-θ distribution map obtained from 5 ns QM/MM MD simulations. b) The average active-site structure of the reactant complex of METTL9 for ethylation in the presence of AdoEth along with r(C_M-N_π)-θ distributions obtained from 5 ns QM/MM MD simulations.

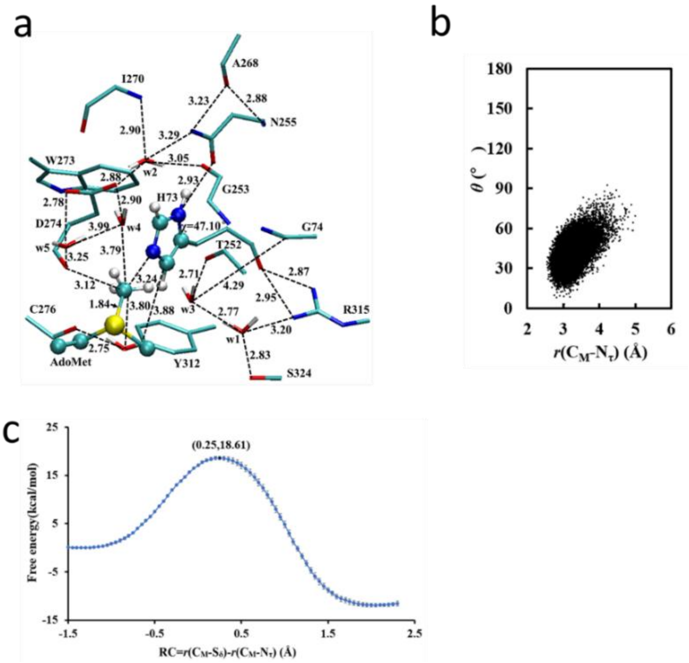

Figure S18. a) The average active-site structure of the reaction state for SETD3 R253G . b) r(CM-Nτ)-θ distribution map. c) The free energy profile for the methyl transfer in SETD3 R253G.

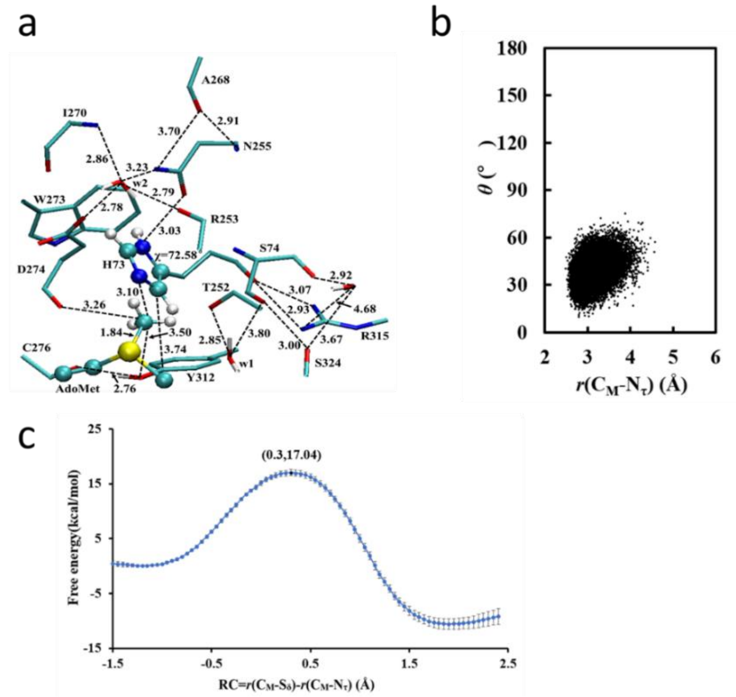

Figure S19. a) The average active-site structure of the reaction state for SETD3 with G74S mutant of βA peptide as the substrate. b) r(CM-Nτ)-θ distribution map. c) The free energy profile for the methyl transfer with G74S mutant of βA peptide.

8. Expression and Purification of SETD3 R253A and R253G variants

Table S1. Sequences of primers used for the site-directed mutagenesis experiments

Primer	Sequence	Protein expressed
R253A-SETD3-S	CTCTTCTGTTATGACG**GCG**CAAAACCAAATTCCCAC	*N*-terminal His_6-tagged R253A mutant of SETD3
R253A-SETD3-AS	GTGGGAATTTGGTTTTG**CGC**CGTCATAACAGAAGAG	
R253G-SETD3-S	CTCTTCTGTTATGACG**GGG**CAAAACCAAATTCCCAC	*N*-terminal His_6-tagged R253G mutant of SETD3
R253G-SETD3-AS	GTGGGAATTTGGTTTTG**CCC**CGTCATAACAGAAGAG	

The nucleotides corresponding to the coding sequences are in capital letters and mutated codons are shown in boldface blue type.

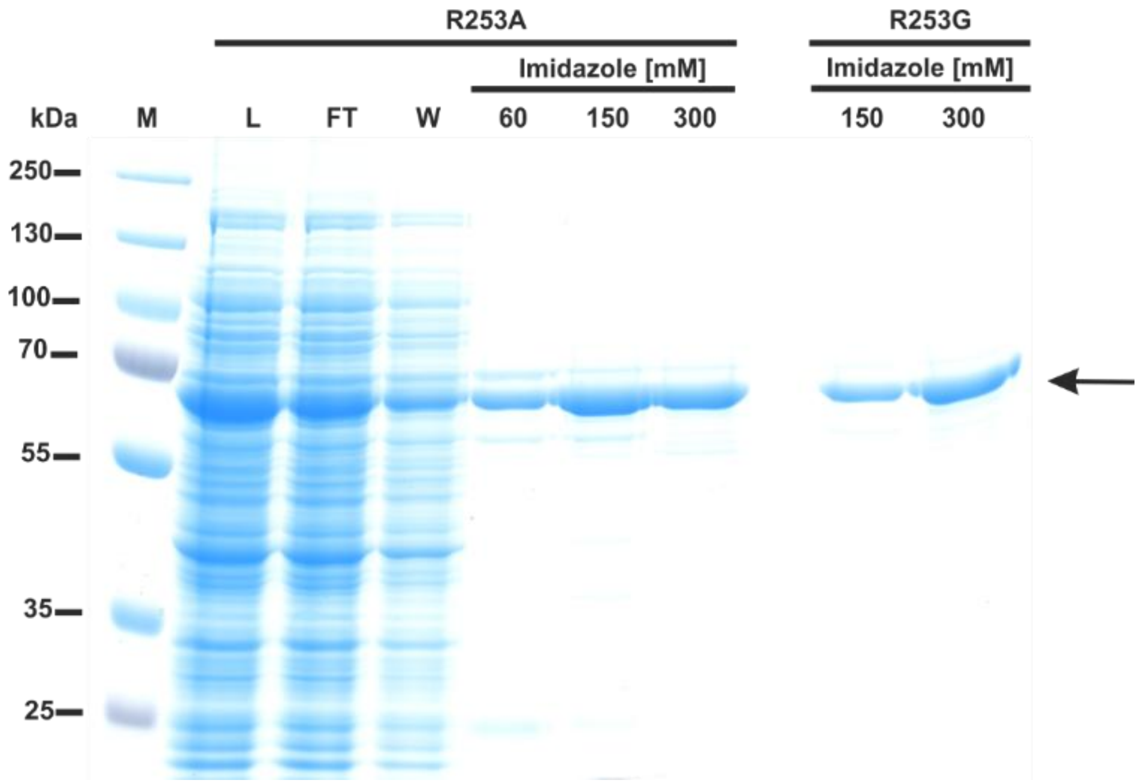

Figure S20. SDS-PAGE analysis of purified recombinant variants R253A and R253G of human SETD3. The recombinant variants R253A and R253G were purified by affinity chromatography using nickel sepharose (HisTrap FF crude), as detailed in the 'Materials and Methods' section. For SDS-PAGE analysis, 11 µl of each eluted fraction was loaded onto a 12% polyacrylamide gel, electrophoresed, and the gel was stained with colloidal Coomassie Brilliant Blue. Analysis of each fraction was performed for variant R253A, while only fractions eluted with the highest concentrations of imidazole and containing the purified enzyme are shown for variant R253G. The arrow indicates protein bands corresponding to the purified R253A or R253G protein. M, prestained protein marker; L, column-loaded cell-free lysate of *E. coli*; FT, flow through; W, wash; Fractions 60 to 300 were eluted with the indicated concentrations of imidazole.
